# Supplementary material for: Power-law scaling of mitotic spindles with genome sizes across eukaryotes is driven by chromosome crowding
Source: Nat Cell Biol. 2026 Oct 9;28(10):1996–2007. doi: 10.1038/s41556-026-02005-8 (PMC13661270; doi:10.1038/s41556-026-02005-8)
Supplement: Supplementary file 1 [file 41556_2026_2005_MOESM1_ESM.pdf]

nature portfolio

Corresponding author(s): Iva M. Tolić and Nenad Pavin

Last updated by author(s): Apr 29, 2026

Reporting Summary

Nature Portfolio wishes to improve the reproducibility of the work that we publish. This form provides structure for consistency and transparency in reporting. For further information on Nature Portfolio policies, see our Editorial Policies and the Editorial Policy Checklist.

Statistics

For all statistical analyses, confirm that the following items are present in the figure legend, table legend, main text, or Methods section.

n/a	Confirmed	
☐	☒	The exact sample size (*n*) for each experimental group/condition, given as a discrete number and unit of measurement
☐	☒	A statement on whether measurements were taken from distinct samples or whether the same sample was measured repeatedly
☐	☒	The statistical test(s) used AND whether they are one- or two-sided<br>*Only common tests should be described solely by name; describe more complex techniques in the Methods section.*
☒	☐	A description of all covariates tested
☐	☒	A description of any assumptions or corrections, such as tests of normality and adjustment for multiple comparisons
☐	☒	A full description of the statistical parameters including central tendency (e.g. means) or other basic estimates (e.g. regression coefficient) AND variation (e.g. standard deviation) or associated estimates of uncertainty (e.g. confidence intervals)
☐	☒	For null hypothesis testing, the test statistic (e.g. *F*, *t*, *r*) with confidence intervals, effect sizes, degrees of freedom and *P* value noted<br>*Give P values as exact values whenever suitable.*
☒	☐	For Bayesian analysis, information on the choice of priors and Markov chain Monte Carlo settings
☒	☐	For hierarchical and complex designs, identification of the appropriate level for tests and full reporting of outcomes
☒	☐	Estimates of effect sizes (e.g. Cohen's *d*, Pearson's *r*), indicating how they were calculated

Our web collection on statistics for biologists contains articles on many of the points above.

Software and code

Policy information about availability of computer code

Data collection	Opterra confocal movies and images were acquired with Prairie View Imaging 5.4.64.500 software (Bruker).<br>STED movies and images were acquired with Imspector 16.3.16118-w2224-win64 software (Abberior Instruments).<br>Airyscan Zeiss LSM800 confocal movies were aquired with ZEN blue 3.5 software (Zeiss).<br>The Lattice Lightsheet 7 system movies were aquired with ZEN 3.7 software (Zeiss).<br>The Observer.Z1 system movies were aquired with SlideBook 5.1 software (Zeiss).<br>Dragonfly spinning disk system movies were aquired with Fusion 2.6 (Andor Technologies).
Data analysis	We used MATLAB R2021a, MATLAB 2019b, Octave 9.2.0 and Python 3.12.9 software for data plotting and calculation of statistical parameters.<br>All movies and images were analyzed with Image J/Fiji.<br>Figures and schematic representations were assembled in Adobe Illustrator 2023 27.9 (Adobe Systems).<br>The code for the numerical computations of the theoretical model is available without restrictions at https://github.com/matkec0/spindleFEM and at https://doi.org/10.5281/zenodo.19708772.

For manuscripts utilizing custom algorithms or software that are central to the research but not yet described in published literature, software must be made available to editors and reviewers. We strongly encourage code deposition in a community repository (e.g. GitHub). See the Nature Portfolio guidelines for submitting code & software for further information.

Data

Policy information about availability of data

All manuscripts must include a data availability statement. This statement should provide the following information, where applicable:

- Accession codes, unique identifiers, or web links for publicly available datasets
- A description of any restrictions on data availability
- For clinical datasets or third party data, please ensure that the statement adheres to our policy

Data on metaphase plate width, thickness and spindle length, width which were reanalyzed in this study can be found in previously published papers [21-23, 43, 50, 52-58, 73, 100-102, 120-123, 125, 127, 128-135, 137-140] together with genome size datasets (GenBank and Animal genome size database) were summarized in Supplementary Table 1-2. The source data used for the main and extended data figures have been deposited in the Figshare database (https://doi.org/10.6084/m9.figshare.32112142)141. All other data supporting the findings of this study are available from the corresponding author.

Research involving human participants, their data, or biological material

Policy information about studies with human participants or human data. See also policy information about sex, gender (identity/presentation), and sexual orientation and race, ethnicity and racism.

Reporting on sex and gender	N/A
Reporting on race, ethnicity, or other socially relevant groupings	N/A
Population characteristics	N/A
Recruitment	N/A
Ethics oversight	N/A

Note that full information on the approval of the study protocol must also be provided in the manuscript.

Field-specific reporting

Please select the one below that is the best fit for your research. If you are not sure, read the appropriate sections before making your selection.

☒ Life sciences ☐ Behavioural & social sciences ☐ Ecological, evolutionary & environmental sciences

For a reference copy of the document with all sections, see nature.com/documents/nr-reporting-summary-flat.pdf

Life sciences study design

All studies must disclose on these points even when the disclosure is negative.

Sample size	Sample size was determined to provide a sufficient statistical power. Sample size and the number of biological replicates for each experiment are indicated in the figure captions.
Data exclusions	Only bipolar spindles were analyzed. Other exclusions were defined in the methods section.
Replication	Experiments were replicated in at least 3 independent experiments.
Randomization	Within each experimental regime, cell populations were assigned randomly to a siRNA, drug or control treatment. No other randomization was used.
Blinding	The investigators were not blinded to allocation during experiments and outcome evaluation.

Reporting for specific materials, systems and methods

We require information from authors about some types of materials, experimental systems and methods used in many studies. Here, indicate whether each material, system or method listed is relevant to your study. If you are not sure if a list item applies to your research, read the appropriate section before selecting a response.

Materials & experimental systems

n/a	Involved in the study
☐	☒ Antibodies
☐	☒ Eukaryotic cell lines
☒	☐ Palaeontology and archaeology
☐	☒ Animals and other organisms
☒	☐ Clinical data
☒	☐ Dual use research of concern
☒	☐ Plants

Methods

n/a	Involved in the study
☒	☐ ChIP-seq
☒	☐ Flow cytometry
☒	☐ MRI-based neuroimaging

Antibodies

Antibodies used

Primary antibodies: anti-α-tubulin (1:500, Sigma) and anti-γ-tubulin antibody (1:1000, Santa Cruz), rabbit anti-centrin-3 (1:300, Abcam, ab228690) and rat anti-tubulin (1:100, MA1-80017, Invitrogen). Secondary antibodies used were: donkey anti-rabbit Alexa Fluor 488 or 594 (1:500, Abcam, ab150061, ab150064, respectively), donkey anti-rabbit Alexa Fluor 647 (1:1000, Abcam, ab150075) and donkey anti-rat Alexa Fluor 594 (1:500, Abcam, ab150156).

Validation

All used antibodies are commercially available and validated before.
-rabbit anti-centrin-3 primary antibody (ab228690, Abcam) is validated for IF by the manufacturer and cited in 1 ref as reported on the manufacturer website (https://www.abcam.com/en-us/products/primary-antibodies/centrin-3-antibody-ab228690), Species Reactivity: human
- rat anti-tubulin (1:500, MA1-80017, Invitrogen) is validated for IF by the manufacturer and cited in 71 refs as reported on the manufacturer website (https://www.thermofisher.com/antibody/product/alpha-Tubulin-Antibody-clone-YL1-2-Monoclonal/MA1-80017), Species Reactivity: dog, fruit fly, human, mouse, plant, pig, rabbit, rat, Xenopus, yeast
- mouse anti-a-tubulin (1:500, Sigma, CP06) is validated for IF by the manufacturer and cited in 658 refs as reported on the manufacturer website (https://www.sigmaaldrich.com/HR/en/product/mm/cp06), Species Reactivity: human, mouse, gerbil, chicken, rat
- goat anti-γ-tubulin (1:1000, Santa Cruz, sc-7396-R) is validated for IF by the manufacturer and cited in 68 refs as reported on the manufacturer website (https://www.scbt.com/de/p/gamma-tubulin-antibody-c-20), Species Reactivity: human
- donkey anti-rat Alexa Fluor 594 (Abcam, ab150156) is validated for IF by the manufacturer and cited in 35 refs as reported on the manufacturer website (https://www.abcam.com/en-us/products/secondary-antibodies/donkey-rat-igg-h-l-alexa-fluor-594-preadsorbed-ab150156)
- donkey anti-rabbit Alexa Fluor 594 (Abcam, ab150076) is validated for IF by the manufacturer and cited in 153 refs as reported on the manufacturer website (https://www.abcam.com/en-us/products/secondary-antibodies/donkey-rabbit-igg-h-l-alexa-fluor-594-ab150076)
- donkey anti-rabbit Alexa Fluor 647 (Abcam, ab150075) is validated for IF by the manufacturer and cited in 489 refs as reported on the manufacturer website (https://www.abcam.com/en-us/products/secondary-antibodies/donkey-rabbit-igg-h-l-alexa-fluor-647-ab150075)
- donkey anti-rabbit Alexa Fluor 488 (Abcam, ab150061) is validated for IF by the manufacturer and cited in 97 refs as reported on the manufacturer website (https://www.abcam.com/en-us/products/secondary-antibodies/donkey-rabbit-igg-h-l-alexa-fluor-488-preadsorbed-ab150061?srsltid=AfmBOoqxjTMqeORzSXbEfqGJiFkUCbtxxPqmNxoyMdvp1apDOwem_Ff9)
- goat anti-mouse DyLight 594 (Abcam, ab96873) is validated for IF by the manufacturer and cited in 24 refs as reported on the manufacturer website (https://www.abcam.com/en-us/products/secondary-antibodies/goat-mouse-igg-h-l-dylight-594-ab96873?srsltid=AfmBOoqt8GIzdkj1x7wnU-1dvlgoedccDsUGmKC4fXQROFNQF5dPCBOo)
- goat anti-rabbit DyLight 650 (Abcam, ab96886) is validated for IF by the manufacturer and cited in 9 refs as reported on the manufacturer website (https://www.abcam.com/en-us/products/secondary-antibodies/goat-rabbit-igg-h-l-dylight-650-ab96886?srsltid=AfmBOorNAgaYIHg_JRcGiMhVTb7NaR926T5cwO9dhPYdauYscKkCB7RQ)

Eukaryotic cell lines

Policy information about cell lines and Sex and Gender in Research

Cell line source(s)

1. human hyper-triploid (modal chromosome number = 74) U2OS (human osteosarcoma, female) cells expressing CENP-A-GFP and mCherry-alpha-tubulin, which were a gift from Helder Maiato (Institute for Molecular Cell Biology, University of Porto, Portugal),
2. human diploid (modal chromosome number = 46) hTERT-RPE1 (retinal pigmented epithelium, female) cells expressing CENP-A-GFP and centrin1-GFP, which were a gift from Alexey Khodjakov (Wadsworth Center, New York State Department of Health, Albany, NY)
3. human diploid hTERT-RPE1 cells and their hypo-tetraploid adapted clones, RPT1, RPT3 and RPT4 (all with modal chromosome number = 80) expressing H2B-GFP (Kuznetzova et. al, 2015.).
4. human diploid hTERT-RPE1 cells with or without p53 knockdown (KD) expressing H2B-Dendra2, which were a gift from Rene Medema (Netherlands Cancer Institute, Amsterdam, Netherlands),
5. Human near-diploid (modal chromosome number = 45) HCT116 (human colorectal carcinoma cell line, male) cells32, and their hypo-tetraploid adapted clones HPT1 (modal chromosome number = 75) and HPT2 (modal chromosome number = 78) (Kuznetzova et. al, 2015.).

Authentication

None of the cell lines was authenticated by the authors.

Mycoplasma contamination	Cells were tested regularly for Mycoplasma contamination using DAPI and were found to be negative for Mycoplasma contamination.
Commonly misidentified lines (See ICLAC register)	No commonly misidentified lines were used in this study.

Animals and other research organisms

Policy information about studies involving animals; ARRIVE guidelines recommended for reporting animal research, and Sex and Gender in Research

Laboratory animals	Mouse small intestine (mSI) organoids were derived from the small intestines of female wild-type C57BL/6 mice, sourced from the Laboratory for Neurodegenerative Disease Research at the Ruđer Bošković Institute, in compliance with the EU Directive 2010/63/EU for animal experimentation and the applicable national regulations of Croatia. The mSI organoids, genetically modified for stable expression of eGFP-Centrin-2, were established using small intestines obtained from mice resulting from crossing of transgenic GFP-CETN2 mice (JAX stock #008234, The Jackson Laboratory) and transgenic Cre-inducible H2B-mCherry mice (JAX stock #0231239, The Jackson Laboratory).
Wild animals	N/A
Reporting on sex	N/A
Field-collected samples	N/A
Ethics oversight	We have complied with all relevant ethical regulations and include a statement affirming this in the manuscript. No ethical approval or guidance of the study protocol was required.

Note that full information on the approval of the study protocol must also be provided in the manuscript.

Plants

Seed stocks	N/A
Novel plant genotypes	N/A
Authentication	N/A
