# Supplementary material for: Power-law scaling of mitotic spindles with genome sizes across eukaryotes is driven by chromosome crowding
Source: Nat Cell Biol. 2026 Oct 9;28(10):1996–2007. doi: 10.1038/s41556-026-02005-8 (PMC13661270; doi:10.1038/s41556-026-02005-8)
Supplement: Supplementary file 2 [file 41556_2026_2005_MOESM2_ESM.pdf]

nature portfolio

Peer Review File

Power-law scaling of mitotic spindles with genome sizes across eukaryotes is driven by chromosome crowding

Corresponding Author: Professor Iva Tolić

Version 0:

Decision Letter:

*Please delete the link to your author homepage if you wish to forward this email to co-authors.

Dear Professor Tolić,

I apologize for the delay.

Your manuscript, "A universal scaling law for mitotic spindles across eukaryotes driven by chromosome crowding", has now been seen by 3 referees, who are experts in MT spindle and size (referee 1); chromatin dynamics and aneuploidy (referee 2); and cytoskeletal dynamics and modelling (referee 3). As you will see from their comments (attached below) they find this work of potential interest, but have raised substantial concerns, which in our view would need to be addressed with considerable revisions before we can consider publication in Nature Cell Biology.

Nature Cell Biology editors discuss the referee reports in detail within the editorial team, including the chief editor, to identify key referee points that should be addressed with priority, and requests that are overruled as being beyond the scope of the current study. To guide the scope of the revisions, I have listed these points below. We are committed to providing a fair and constructive peer-review process, so please feel free to contact me if you would like to discuss any of the referee comments further.

In particular, it would be essential to:

A) Justify modelling parameters (all Reviewers)

B) Test effects of ploidy and cytoplasmic density (Reviewer #2)

C) Address concerns about force vectors and effects on microtubules and chromosomes (Reviewers #2 and #3)

D) All other referee concerns pertaining to strengthening existing data, providing controls, methodological details, clarifications and textual changes, should also be addressed.

E) Finally please pay close attention to our guidelines on statistical and methodological reporting (listed below) as failure to do so may delay the reconsideration of the revised manuscript. In particular please provide:

- a Supplementary Figure including unprocessed images of all gels/blots in the form of a multi-page pdf file. Please ensure that blots/gels are labeled and the sections presented in the figures are clearly indicated.

- a Supplementary Table including all numerical source data in Excel format, with data for different figures provided as different sheets within a single Excel file. The file should include source data giving rise to graphical representations and statistical descriptions in the paper and for all instances where the figures present representative experiments of multiple independent repeats, the source data of all repeats should be provided.

We would be happy to consider a revised manuscript that would satisfactorily address these points, unless a similar paper is published elsewhere, or is accepted for publication in Nature Cell Biology in the meantime.

When revising the manuscript please:

- ensure that it conforms to our format instructions and publication policies (see below and https://www.nature.com/nature/for-authors).

- provide a point-by-point rebuttal to the full referee reports verbatim, as provided at the end of this letter.

- provide the completed Reporting Summary (found here https://www.nature.com/documents/nr-reporting-summary.pdf). This is essential for reconsideration of the manuscript will be available to editors and referees in the event of peer review. For more information see http://www.nature.com/authors/policies/availability.html or contact me.

When submitting the revised version of your manuscript, please pay close attention to our href="https://www.nature.com/nature-portfolio/editorial-policies/image-integrity">Digital Image Integrity Guidelines. and to the following points below:

-- that unprocessed scans are clearly labelled and match the gels and western blots presented in figures.
-- that control panels for gels and western blots are appropriately described as loading on sample processing controls
-- all images in the paper are checked for duplication of panels and for splicing of gel lanes.

Finally, please ensure that you retain unprocessed data and metadata files after publication, ideally archiving data in perpetuity, as these may be requested during the peer review and production process or after publication if any issues arise.

Nature Cell Biology is committed to improving transparency in authorship. As part of our efforts in this direction, we are now requesting that all authors identified as ‘corresponding author’ on published papers create and link their Open Researcher and Contributor Identifier (ORCID) with their account on the Manuscript Tracking System (MTS), prior to acceptance. ORCID helps the scientific community achieve unambiguous attribution of all scholarly contributions. You can create and link your ORCID from the home page of the MTS by clicking on ‘Modify my Springer Nature account’. For more information please visit please visit www.springernature.com/orcid.

This journal strongly supports public availability of data. Please place the data used in your paper into a public data repository, or alternatively, present the data as Supplementary Information. If data can only be shared on request, please explain why in your Data Availability Statement, and also in the correspondence with your editor. Please note that for some data types, deposition in a public repository is mandatory - more information on our data deposition policies and available repositories appears below.

Please submit the revised manuscript files and the point-by-point rebuttal to the referee comments using this link:

Link Redacted

*This url links to your confidential home page and associated information about manuscripts you may have submitted or be reviewing for us. If you wish to forward this email to co-authors, please delete the link to your homepage.

We would like to receive a revised submission within six months.

We hope that you will find our referees' comments, and editorial guidance helpful. Please do not hesitate to contact me if there is anything you would like to discuss.

Best wishes,

Daryl

Daryl Jason Verzosa David, PhD

Senior Editor, Nature Cell Biology
Advisory Editor, npj Biological Physics and Mechanics
Nature Portfolio

Heidelberger Platz 3, 14197 Berlin, Germany
Email: daryl.david@nature.com
ORCID: https://orcid.org/0000-0002-9253-4805

Reviewers' Comments:

Reviewer #1 (Remarks to the Author):

A. Summary of the key results:
The abstract accurately summarizes the paper
B. Originality and significance:
The study is novel and interesting. There has been a lot of interest in spindle scaling, but published studies focused on reduction divisions in early embryos. This paper focuses on a more general problem, scaling of somatic cell spindles across species. This allows the authors to investigate a large range of genome sizes. The study encompasses plants and microorganisms, which is very nice from a significance perspective.
C. Data & methodology: validity of approach, quality of data, quality of presentation:
Overall good, but I feel the paper could lay out the conceptual arguments more clearly for a general audience, esp. early in the results. Finding that a linear dimension of an object scales as the cube root of its mass is the expected result if the object keeps a constant shape as its mass changes. The authors find that metaphase width indeed increases as the cube root of mass, but spindle width does not. This means that the shape and/or density are not constant. Figure 1 should include an estimate of density based on length and width and perhaps a shape parameter also. Estimated density is an important part of the results and showing it explicitly would help readers understand the problem that needs to be solved using a mechanical model. I like figure 5. It lays out the problem more simply than figure 3. I think it should come earlier in the paper, before figure 3, as a conceptual basis that example the problem that requires a mechanical model to solve.
D. Appropriate use of statistics and treatment of uncertainties:
OK
E. Conclusions: robustness, validity, reliability:
Strong, though as detailed above the authors should include a specific estimate of DNA density in the metaphase plate, inferred from width and length, and use that to both explain why a mechanical model is needed and how it solves the non-constant density part of the problem.
F. Suggested improvements: experiments, data for possible revision:
As detailed above. No new experiments are needed. This is a v interesting paper. The data are very simple. But I feel the conceptual layout of the paper can be improved
G. References: appropriate credit to previous work?:
OK
H. Clarity and context: lucidity of abstract/summary, appropriateness of abstract, introduction and conclusions:
OK, but see comments on conceptual argument as detailed above.

Reviewer #2 (Remarks to the Author):

Accurate chromosome segregation during mitosis requires the precise organization of chromosomes within the spindle apparatus. While previous studies have examined how spindle size scales with cell and nuclear dimensions, the influence of genome size and chromosome number on spindle architecture remains less well understood.
This study presents a universal scaling law that links metaphase plate width and spindle width to genome size and chromosome number across diverse eukaryotic systems. Using a physical model and compression experiments on mitotic cells, the authors elucidate how inter-chromosomal pushing forces shape spindle morphology. They propose that chromosome crowding plays a central role in determining metaphase plate width/spindle width, whereas spindle length is likely governed by independent mechanisms, such as the activity of microtubule-associated proteins. These findings deepen our understanding of the biophysical principles that govern cell division in eukaryotes, and they are of interest to NCB readers. However, several critical issues must be addressed before the manuscript is suitable for publication.

Major remarks:
1. In Box 1, the formula F=–ch3/2 likely derives from the Hertz contact model. The exponent 3/2 is critical for their model and may dominate the resulting power law exponent. The authors should clarify why this model was chosen, how biologically relevant it is (including assumptions), and whether alternative models were tested or considered.
2. In Fig. 1, the authors show that metaphase plate width correlates with genome size, but not with chromosome number. How do they explain this? This referee would like to see how plate width scales with genome size when chromosome number is held constant, and conversely, how it scales with chromosome number when genome size is held constant, across different eukaryotes.
3. In Fig. 2, it's unclear why the authors switch from genome size to interphase nuclear area. How do the authors use interphase nuclear area as a proxy for chromosome number? In Extended Data Fig. 4d, nuclear area does not increase with chromosome number, which contradicts their claim. This reviewer agrees that nuclear area generally reflects ploidy number, making it a useful proxy for estimating ploidy. However, the relationship is not perfectly fixed and can be influenced by cell type and chromatin state. Nuclear volume is a more precise metric than nuclear area for this purpose. Since the nucleus has a 3D shape, its total volume—not just area—better reflects chromosome number.
4. For organoids, how were the target cells selected? Could the nucleus or spindle be compressed by neighboring cells in PDOs, as shown in Fig. 4? The authors also need to explain what the "24Ta, 24Tb, 9T…" cell types refer to, and how they define lower or higher ploidy. For panels e and c, consider using log-log plots.
5. In Fig. 2, another way to reflect pushing forces between chromosomes at the metaphase plate is by measuring their DNA intensity. According to the authors' model, higher crowding and pushing forces would suggest greater average intensity. The authors should measure DNA intensity across different ploidy levels, MUG cells, and compression conditions.
6. Since spindle size has been shown to scale with cell size, have the authors measured cell size across different ploidy levels? This might explain why spindle length remains unchanged in MUG cells.
7. In Fig. 2, metaphase plate thickness appears to vary with ploidy or chromosome number. However, why is there no difference in metaphase plate width/thickness between hypotetraploid cells and tetraploid cells, despite genome density

being different? Can the authors comment on that?
8. In Figs. 2h/i and Extended Figs. 4f/g, Cenp-E–depleted chromosomes on the metaphase plate appear more dispersed. However, based on their model, they should be more compacted due to their reduced size or stiffness. How do the authors explain this discrepancy? Also, spindle lengths in these cells appear longer—could this be an adaptation to maintain global force balance? If so, does this imply that spindle length is indirectly affected by chromosome number?
9. In Extended Fig. 3, the authors report a correlation between plate thickness and interphase nuclear area in both RPE1 and PDO cells. Since metaphase plate volume is proportional to W^2×T (where W is width and T is thickness), both parameters should be included in the model. Accordingly, the authors should complete the metaphase thickness dataset and consider updating their model—perhaps using a multilayer elastic-ball approach—to incorporate thickness.
10. In Fig. 3, the authors state: "We use spheres as the simplest geometric representation of chromosome shape, taking their volume as a proxy for DNA content." The authors should clarify why and how they chose elastic spheres, referencing studies 51–53. Since they use DNA content in their calculations, they clearly need to define this term along with their assumptions (see also your first minor comment).
11. In Figs. 3b/c, the authors infer that the scaling exponent between W and V is 1/3, and between W and N is 1/2. However, these conclusions are incomplete without accounting for plate thickness, as noted in comment 9.
12. In their model, have the authors considered the role of cytoplasmic forces that could push mitotic chromosomes via crowding? Osmotic shock experiments could help test this. Additionally, could microtubules themselves contribute to chromosome pushing? Please clarify.

13. In Fig. 4, this reviewer is not fully convinced by their compression experiments. While well-performed, they do not clearly address their central hypothesis. After compression along the Y-axis, it is unclear whether inter-chromosome pushing forces along the Z-axis increase. Therefore, concluding that Wz expands due to inter-chromosome forces may not be justified. Moreover, even if chromosomes are not in contact on the spindle (e.g., only the two outer ones are), the plate width can still expand upon compression due to the elastic properties of microtubules, which behave like springs. A different or more direct experiment would better support the model.
14. In Fig. 5, they claim that when DNA length increases by a factor of n, chromosome volume increases n-fold—this holds only if DNA density remains constant. Similarly, chromosome length increases by n^1/3 only if the shape remains constant (which is probably not the case in reality). The same logic applies to metaphase plate width: it increases with n^1/3 only if plate thickness scales in the same way. The authors should state all their assumptions clearly and explain how they diverge from biological reality.
15. The authors stated: "Based on these results, we conclude that substantially larger inter-chromosome pushing forces impede mitosis." Does the spindle fully recover its shape after compression is released? Is the recovery dependent on the duration or strength of compression? If the spindle was distorted or the DNA damaged during compression, that might explain the observed delay in metaphase. In such cases, did they observe any chromosome mis-segregation? Specifically, from Fig. 6i: can the authors separate the cells that return to normal from those that do not, and check mis-segregation frequency?

Minor remarks:
a) In the text, please pay attention to the terms used-chromosome number, genome size, nuclear area, amount of chromatin, and DNA content. Define each term clearly and specify the assumptions made regarding their relationships.
b) Please add statistical correlation directly in the graph of figure 1. Why is the statistical correlation analysis different between 1d and e?
c) Figure 2b: why does cell number different between up and bottom? Also the number of cells analysed for this figure is quite limited. What is the limiting factor?
d) The figures could be better organized. For example, Fig. 2c should come before Fig. 2d, and Fig. 4e should be placed alongside the corresponding experimental analysis.
e) Figure 2h: the MUG image is not very convincing and the cell number is very low (~12). It should be replaced by a clearer image and the sample analysed increase extensively.
f) In Fig. 3e, add the line of w=15 µm (yellow) to facilitate comparison across conditions.
g) On Page 3, the authors wrote: "We infer that the total amount of chromatin in the metaphase plate, rather than the chromosome number and the spindle length, is at the core of the spindle adaptation mechanism." This reviewer assume that "amount of chromatin" refers to genome size. However, the authors cannot rule out spindle length as a contributing factor, as Fig. 1e shows that spindle length also correlates with genome size. Moreover, although the authors suggest that chromosome number is not central, they later show that plate width scales with interphase area /chromosome number (Fig. 2), which contradicts this claim.

Reviewer #3 (Remarks to the Author):

In this very interesting paper, the authors measure the scaling between the genome size and the metaphase plate width, and explain the scaling with a mechanical model based on the idea that the chromosomes are compressed by mechanical forces. The reported scaling is novel, but I find the model completely confusing and unconvincing, and the scaling is so simple that it probably could be explained by several other ideas.

1) The 'spheres' - are those individual chromosomes? - because the individual chromosomes are obviously not spherical in the metaphase plate, as is apparent from many published images.

2) It seems that in the model the chromosomes are pressed centripetally inward toward the spindle axis, correct? But then this force is generated by MT pulling? It does not look like this pulling force was considered in the MT elastic rod equation. If MTs are pulling, why do they have the curved shape? What are the boundary conditions at the poles and on chromosomes? Some MTs are KT-MTs, some are chromosomal. The former are supposed to be pulling, the latter - pushing. Comments on this complication?

3) The main problem: if indeed in the model the chromosomes are pressed centripetally inward toward the spindle axis, why would not the metaphase plate buckle? - why would not the inner chromosomes be pushed out away from the equator?

4) Presumably, as the forces are MT-related, the scaling should depend on the MT number. Was this researched?

Additional questions and comments:

Are the considered spindle cylindrically symmetric? - does the metaphase plate have a cylindrical shape? If not, I am not sure what was measured in the paper, and in adherent cells (and also in tissue cells) spindles are rarely cylindrically symmetric...

Similarly, when the spindles were deformed - in which direction? And if the deformed spindles became asymmetric, how was the theory used?

In fig 1, it is apparent that the difference between the metaphase plate width and spindle width are just 'rabbit-ears' of the chromosome arms, which are quite obviously not cylindrical... Comments?

Presumably, one can compute the total chromosome volume from the metaphase plate thickness and width - and then compare it with the nuclear volume - this will be a good sanity check. Was this done? What is the relation between thickness and width?

Lastly, a paper on spindle scaling cannot omit citing the following two fundamental papers (I am, by the way, not one of these papers' co-authors and have no hidden agenda :)) and discussing what your study implies for their results:

Stoichiometric interactions explain spindle dynamics and scaling across 100 million years of nematode evolution
Reza Farhadifar et al, eLife 2020

Curr Biol. 2015 Mar 16;25(6):732-740. doi: 10.1016/j.cub.2014.12.060. Epub 2015 Feb 12.
Scaling, selection, and evolutionary dynamics of the mitotic spindle
Reza Farhadifar et al

Similarly, this review lists all possible mechanisms of the spindle scaling:

Curr Biol. 2009 Sep 15;19(17):R749-61. doi: 10.1016/j.cub.2009.07.028.
Force and length in the mitotic spindle
Sophie Dumont, Timothy J Mitchison

It'd be good to cite it (again, I am not one of the authors) and discuss.

I realize of course that your focus is not on the spindle length but on the metaphase plate width.

Reviewer #3 (Remarks on Protocol(s)):

I am not a great expert on experiment, but all seems to be in order.
The model, as I wrote, I found confusing - could be just the problem with explaining it better - I know the authors' previous work, and it is excellent

GUIDELINES FOR MANUSCRIPT SUBMISSION TO NATURE CELL BIOLOGY

READABILITY OF MANUSCRIPTS – Nature Cell Biology is read by cell biologists from diverse backgrounds, many of whom are not native English speakers. Authors should aim to communicate their findings clearly, explaining technical jargon that might be unfamiliar to non-specialists, and avoiding non-standard abbreviations. Titles and abstracts should concisely communicate the main findings of the study, and the background, rationale, results and conclusions should be clearly explained in the manuscript in a manner accessible to a broad cell biology audience. Nature Cell Biology uses British spelling.

MANUSCRIPT FORMAT – please follow the guidelines listed in our Guide to Authors regarding manuscript formats at Nature Cell Biology.

TITLE – should be no more than 100 characters including spaces, without punctuation and avoiding technical terms, abbreviations, and active verbs..

AUTHOR NAMES – should be given in full.

AUTHOR AFFILIATIONS – should be denoted with numerical superscripts (not symbols) preceding the names. Full addresses should be included, with US states in full and providing zip/post codes. The corresponding author is denoted by: "Correspondence should be addressed to [initials]."

ABSTRACT AND MAIN TEXT – please follow the guidelines that are specific to the format of your manuscript, as listed in our Guide to Authors (http://www.nature.com/ncb/pdf/ncb_gta.pdf) Briefly, Nature Cell Biology Articles, Resources and Technical Reports have 3500 words, including a 150 word abstract, and the main text is subdivided in Introduction, Results, and Discussion sections. Nature Cell Biology Letters have up to 2500 words, including a 180 word introductory paragraph (abstract), and the text is not subdivided in sections.

ACKNOWLEDGEMENTS – should be kept brief. Professional titles and affiliations are unnecessary. Grant numbers can be listed.

AUTHOR CONTRIBUTIONS – must be included after the Acknowledgements, detailing the contributions of each author to the paper (e.g. experimental work, project planning, data analysis etc.). Each author should be listed by his/her initials.

FINANCIAL AND NON-FINANCIAL COMPETING INTERESTS – the authors must include one of three declarations: (1) that they have no financial and non-financial competing interests; (2) that they have financial and non-financial competing interests; or (3) that they decline to respond, after the Author Contributions section. This statement will be published with the article, and in cases where financial and non-financial competing interests are declared, these will be itemized in a web supplement to the article. For further details please see https://www.nature.com/licenceforms/nrg/competing-interests.pdf.

REFERENCES – are limited to a total of 70 for Articles, Resources, Technical Reports; and 40 for Letters. This includes references in the main text and Methods combined. References must be numbered sequentially as they appear in the main text, tables and figure legends and Methods and must follow the precise style of Nature Cell Biology references. References only cited in the Methods should be numbered consecutively following the last reference cited in the main text. References only associated with Supplementary Information (e.g. in supplementary legends) do not count toward the total reference limit and do not need to be cited in numerical continuity with references in the main text. Only published papers can be cited, and each publication cited should be included in the numbered reference list, which should include the manuscript titles. Footnotes are not permitted.

METHODS – Nature Cell Biology publishes methods online. The methods section should be provided as a separate Word document, which will be copyedited and appended to the manuscript PDF, and incorporated within the HTML format of the paper.

Methods should be written concisely, but should contain all elements necessary to allow interpretation and replication of the results. As a guideline, Methods sections typically do not exceed 3,000 words. The Methods should be divided into subsections listing reagents and techniques. When citing previous methods, accurate references should be provided and any alterations should be noted. Information must be provided about: antibody dilutions, company names, catalogue numbers and clone numbers for monoclonal antibodies; sequences of RNAi and cDNA probes/primers or company names and catalogue numbers if reagents are commercial; cell line names, sources and information on cell line identity and authentication. Animal studies and experiments involving human subjects must be reported in detail, identifying the committees approving the protocols. For studies involving human subjects/samples, a statement must be included confirming that informed consent was obtained. Statistical analyses and information on the reproducibility of experimental results should be provided in a section titled “Statistics and Reproducibility”.

All Nature Cell Biology manuscripts submitted on or after March 21 2016 must include a Data availability statement as a separate section after Methods but before references, under the heading "Data Availability”. . For Springer Nature policies on data availability see http://www.nature.com/authors/policies/availability.html; for more information on this particular policy see http://www.nature.com/authors/policies/data/data-availability-statements-data-citations.pdf. The Data availability statement should include:

• Accession codes for primary datasets (generated during the study under consideration and designated as "primary accessions") and secondary datasets (published datasets reanalysed during the study under consideration, designated as

"referenced accessions"). For primary accessions data should be made public to coincide with publication of the manuscript. A list of data types for which submission to community-endorsed public repositories is mandated (including sequence, structure, microarray, deep sequencing data) can be found here http://www.nature.com/authors/policies/availability.html#data.

• Unique identifiers (accession codes, DOIs or other unique persistent identifier) and hyperlinks for datasets deposited in an approved repository, but for which data deposition is not mandated (see here for details http://www.nature.com/sdata/data-policies/repositories).

• At a minimum, please include a statement confirming that all relevant data are available from the authors, and/or are included with the manuscript (e.g. as source data or supplementary information), listing which data are included (e.g. by figure panels and data types) and mentioning any restrictions on availability.

• If a dataset has a Digital Object Identifier (DOI) as its unique identifier, we strongly encourage including this in the Reference list and citing the dataset in the Methods.

We recommend that you upload the step-by-step protocols used in this manuscript to protocols.io. More details can found at https://www.protocols.io/help/publish-articles.

DISPLAY ITEMS – main display items are limited to 6-8 main figures and/or main tables for Articles, Resources, Technical Reports; and 5 main figures and/or main tables for Letters. For Supplementary Information see below.

FIGURES – Colour figure publication costs $600 for the first, and $300 for each subsequent colour figure. All panels of a multi-panel figure must be logically connected and arranged as they would appear in the final version. Unnecessary figures and figure panels should be avoided (e.g. data presented in small tables could be stated briefly in the text instead).

All imaging data should be accompanied by scale bars, which should be defined in the legend.
Cropped images of gels/blots are acceptable, but need to be accompanied by size markers, and to retain visible background signal within the linear range (i.e. should not be saturated). The boundaries of panels with low background have to be demarked with black lines. Splicing of panels should only be considered if unavoidable, and must be clearly marked on the figure, and noted in the legend with a statement on whether the samples were obtained and processed simultaneously. Quantitative comparisons between samples on different gels/blots are discouraged; if this is unavoidable, it should only be performed for samples derived from the same experiment with gels/blots were processed in parallel, which needs to be stated in the legend.

Figures should be provided at approximately the size that they are to be printed at (single column is 86 mm, double column is 170 mm) and should not exceed an A4 page (8.5 x 11"). Reduction to the scale that will be used on the page is not necessary, but multi-panel figures should be sized so that the whole figure can be reduced by the same amount at the smallest size at which essential details in each panel are visible. In the interest of our colour-blind readers we ask that you avoid using red and green for contrast in figures. Replacing red with magenta and green with turquoise are two possible colour-safe alternatives. Lines with widths of less than 1 point should be avoided. Sans serif typefaces, such as Helvetica (preferred) or Arial should be used. All text that forms part of a figure should be rewritable and removable.

We accept files from the following graphics packages in either PC or Macintosh format:

- For line art, graphs, charts and schematics we prefer Adobe Illustrator (.AI), Encapsulated PostScript (.EPS) or Portable Document Format (.PDF). Files should be saved or exported as such directly from the application in which they were made, to allow us to restyle them according to our journal house style.

- We accept PowerPoint (.PPT) files if they are fully editable. However, please refrain from adding PowerPoint graphical effects to objects, as this results in them outputting poor quality raster art. Text used for PowerPoint figures should be Helvetica (preferred) or Arial.

- We do not recommend using Adobe Photoshop for designing figures, but we can accept Photoshop generated (.PSD or .TIFF) files only if each element included in the figure (text, labels, pictures, graphs, arrows and scale bars) are on separate layers. All text should be editable in 'type layers' and line-art such as graphs and other simple schematics should be preserved and embedded within 'vector smart objects' - not flattened raster/bitmap graphics.

- Some programs can generate Postscript by 'printing to file' (found in the Print dialogue). If using an application not listed above, save the file in PostScript format or email our Art Editor, Allen Beattie for advice (a.beattie@nature.com).

Regardless of format, all figures must be vector graphic compatible files, not supplied in a flattened raster/bitmap graphics format, but should be fully editable, allowing us to highlight/copy/paste all text and move individual parts of the figures (i.e. arrows, lines, x and y axes, graphs, tick marks, scale bars etc.). The only parts of the figure that should be in pixel raster/bitmap format are photographic images or 3D rendered graphics/complex technical illustrations.

All placed images (i.e. a photo incorporated into a figure) should be on a separate layer and independent from any superimposed scale bars or text. Individual photographic images must be a minimum of 300+ DPI (at actual size) or kept

constant from the original picture acquisition and not decreased in resolution post image acquisition. All colour artwork should be RGB format.

EXTENDED DATA FIGURES - When re-submitting your manuscript, please ensure that any supplementary figures and tables that are crucial to the manuscript's conclusions are converted into Extended Data figures and tables to increase visibility of these data. Extended Data figures and tables are online-only (present in the online PDF and full-text HTML versions of the paper), peer-reviewed display items that provide essential background to the article but are not included in the main article due to space constraints. A maximum of ten Extended Data display items (figures and tables) is permitted.

FIGURE LEGENDS – must not exceed 350 words for each figure to allow fit on a single printed NCB page together with the figure. They must include a brief title for the whole figure, and short descriptions of each panel with definitions of the symbols used, but without detailing methodology.

TABLES – main tables should be provided as individual Word files, together with a brief title and legend. For supplementary tables see below.

SUPPLEMENTARY INFORMATION – Supplementary information is material directly relevant to the conclusion of a paper, but which cannot be included in the printed version in order to keep the manuscript concise and accessible to the general reader. Supplementary information is an integral part of a Nature Cell Biology publication and should be prepared and presented with as much care as the main display item, but it must not include non-essential data or text, which may be removed at the editor's discretion. All supplementary material is fully peer-reviewed and published online as part of the HTML version of the manuscript. Supplementary Figures and Supplementary Notes are appended at the end of the main PDF of the published manuscript.

Supplementary items should relate to a main text figure, wherever possible, and should be mentioned sequentially in the main manuscript, designated as Supplementary Figure, Table, Video, or Note, and numbered continuously (e.g. Supplementary Figure 1, Supplementary Figure 2, Supplementary Table 1, Supplementary Table 2 etc.).

Unprocessed scans of all key data generated through electrophoretic separation techniques need to be presented in a supplementary figure that should be labelled and numbered as the final supplementary figure, and should be mentioned in every relevant figure legend. This figure does not count towards the total number of figures and is the only figure that can be displayed over multiple pages, but should be provided as a single file, in PDF or TIFF format. Data in this figure can be displayed in a relatively informal style, but size markers and the figures panels corresponding to the presented data must be indicated.

The total number of Supplementary Figures (not including the "unprocessed scans" Supplementary Figure) should not exceed the number of main display items (figures and/or tables (see our Guide to Authors and March 2012 editorial http://www.nature.com/ncb/authors/submit/index.html#suppinfo; http://www.nature.com/ncb/journal/v14/n3/index.html#ed). No restrictions apply to Supplementary Tables or Videos, but we advise authors to be selective in including supplemental data.

Each Supplementary Figure should be provided as a single page and as an individual file in one of our accepted figure formats and should be presented according to our figure guidelines (see above). Supplementary Tables should be provided as individual Excel files. Supplementary Videos should be provided as .avi or .mov files up to 50 MB in size. Supplementary Figures, Tables and Videos much be accompanied by a separate Word document including titles and legends.

GUIDELINES FOR EXPERIMENTAL AND STATISTICAL REPORTING

REPORTING REQUIREMENTS – We are trying to improve the quality of methods and statistics reporting in our papers. To that end, we are now asking authors to complete a reporting summary that collects information on experimental design and reagents. The Reporting Summary can be found here https://www.nature.com/documents/nr-reporting-summary.pdf)If you would like to reference the guidance text as you complete the template, please access these flattened versions at http://www.nature.com/authors/policies/availability.html.

STATISTICS – Wherever statistics have been derived the legend needs to provide the n number (i.e. the sample size used to derive statistics) as a precise value (not a range), and define what this value represents. Error bars need to be defined in the legends (e.g. SD, SEM) together with a measure of centre (e.g. mean, median). Box plots need to be defined in terms of minima, maxima, centre, and percentiles. Ranges are more appropriate than standard errors for small data sets. Wherever statistical significance has been derived, precise p values need to be provided and the statistical test used needs to be stated in the legend. Statistics such as error bars must not be derived from n<3. For sample sizes of n<5 please plot the individual data points rather than providing bar graphs. Deriving statistics from technical replicate samples, rather than biological replicates is strongly discouraged. Wherever statistical significance has been derived, precise p values need to be provided and the statistical test stated in the legend.

Information on how many times each experiment was repeated independently with similar results needs to be provided in the legends and/or Methods for all experiments, and in particular wherever representative experiments are shown.

We strongly recommend the presentation of source data for graphical and statistical analyses as a separate Supplementary Table, and request that source data for all independent repeats are provided when representative experiments of multiple independent repeats, or averages of two independent experiments are presented. This supplementary table should be in Excel format, with data for different figures provided as different sheets within a single Excel file. It should be labelled and numbered as one of the supplementary tables, titled "Statistics Source Data", and mentioned in all relevant figure legends.

--------- Please don't hesitate to contact NCB@nature.com should you have queries about any of the above requirements ---------

Version 1:

Decision Letter:

Our ref: NCB-A57858A

18th March 2026

Dear Dr. Tolić,

Thank you for submitting your revised manuscript "A universal scaling law for mitotic spindles across eukaryotes driven by chromosome crowding" (NCB-A57858A). It has now been seen by the original referees and their comments are below. The reviewers find that the paper has improved in revision, and therefore we'll be happy in principle to publish it in Nature Cell Biology, pending minor revisions to satisfy the referees' final requests and to comply with our editorial and formatting guidelines.

If the current version of your manuscript is in a PDF format, please email us a copy of the file in an editable format (Microsoft Word or LaTex)-- we can not proceed with PDFs at this stage.

We are now performing detailed checks on your paper and will send you a checklist detailing our editorial and formatting requirements in about a week. Please do not upload the final materials and make any revisions until you receive this additional information from us.

Thank you again for your interest in Nature Cell Biology Please do not hesitate to contact me if you have any questions.

Sincerely,

Daryl

Daryl Jason Verzosa David, PhD

Senior Editor, Nature Cell Biology
Advisory Editor, npj Biological Physics and Mechanics
Nature Portfolio

Heidelberger Platz 3, 14197 Berlin, Germany
Email: daryl.david@nature.com
ORCID: https://orcid.org/0000-0002-9253-4805

Reviewer #1 (Remarks to the Author):

The authors seem to have done a good jb responding to reviewer concerns

Reviewer #1 (Remarks on code availability):

You cannot expect reviewers to do this kind of work for you. This would be a ton of work!

Reviewer #1 (Remarks on Protocol(s)):

You cannot ask reviewers to do this much work. Ask GPT5 to review, it will do a good job

Reviewer #2 (Remarks to the Author):

The authors did an impressive job addressing all the comments, and the final manuscript is now very solid. I have just one final note regarding comment #7: for clarity, I suggest that the authors include in the Discussion the hypothesis explaining the difference between adaptive hypotetraploid clones and those experimentally induced. This could help justify the lack of

difference in metaphase plate width/thickness between hypotetraploid and tetraploid cells, despite their differing genome densities

Reviewer #3 (Remarks to the Author):

The authors properly addressed all critiques and questions, and the paper is ready for acceptance.

Reviewer #3 (Remarks on code availability):

the equations the authors use are quite easy and transparent and are explained well in the SI

Reviewer #3 (Remarks on Protocol(s)):

I am not an expert on the experimental protocols

Version 2:

Decision Letter:

Dear Dr Tolić,

I am pleased to inform you that your manuscript, "Power-law scaling of mitotic spindles with genome sizes across eukaryotes is driven by chromosome crowding", has now been accepted for publication in Nature Cell Biology.

Thank you for sending us the final manuscript files to be processed for print and online production, and for returning the manuscript checklists and other forms. Your manuscript will now be passed to our production team who will be in contact with you if there are any questions with the production quality of supplied figures and text.

Over the next few weeks, your paper will be copyedited to ensure that it conforms to Nature Cell Biology style. Once your paper is typeset, you will receive an email with a link to choose the appropriate publishing options for your paper and our Author Services team will be in touch regarding any additional information that may be required.

After the grant of rights is completed, you will receive a link to your electronic proof via email with a request to make any corrections within 48 hours. If, when you receive your proof, you cannot meet this deadline, please inform us at rjsproduction@springernature.com immediately.

You will not receive your proofs until the publishing agreement has been received through our system.

Due to the importance of these deadlines, we ask that you please let us know now whether you will be difficult to contact over the next month. If this is the case, we ask you provide us with the contact information (email, phone and fax) of someone who will be able to check the proofs on your behalf, and who will be available to address any last-minute problems.

If you have any questions about our publishing options, costs, Open Access requirements, or our legal forms, please contact ASJournals@springernature.com

Once your paper has been scheduled for online publication, the Nature press office will be in touch to confirm the details. An online order form for reprints of your paper is available at https://www.nature.com/reprints/author-reprints.html. All co-authors, authors' institutions and authors' funding agencies can order reprints using the form appropriate to their geographical region.

Publication is conditional on the manuscript not being published elsewhere and on there being no announcement of this work to any media outlet until the online publication date in Nature Cell Biology.

You may wish to make your media relations office aware of your accepted publication, in case they consider it appropriate to organize some internal or external publicity. Once your paper has been scheduled you will receive an email confirming the publication details. This is normally 3-4 working days in advance of publication. If you need additional notice of the date and time of publication, please let the production team know when you receive the proof of your article to ensure there is sufficient time to coordinate. Further information on our embargo policies can be found here: https://www.nature.com/authors/policies/embargo.html

Please note that *Nature Cell Biology* is a Transformative Journal (TJ). Authors may publish their research with us through the traditional subscription access route or make their paper immediately open access through payment of an article-processing charge (APC). Authors will not be required to make a final decision about access to their article until it has been accepted. Find out more about

Transformative Journals

Authors may need to take specific actions to achieve compliance with funder and institutional open access mandates. If your research is supported by a funder that requires immediate open access (e.g. according to Plan S principles or the NIH public access policy) then you should select the gold OA route, and we will direct you to the compliant route where possible. Because authors warrant under our subscription licensing terms that they haven't committed to licensing any version of their article under a licence inconsistent with the terms of our agreement – including the applicable embargo period – publication under the subscription model isn't suitable for authors whose funders require no embargo.

To assist our authors in disseminating their research to the broader community, our SharedIt initiative provides you with a unique shareable link that will allow anyone (with or without a subscription) to read the published article. Recipients of the link with a subscription will also be able to download and print the PDF.

If your paper includes color figures, please be aware that in order to help cover some of the additional cost of four-color reproduction, Nature Portfolio charges our authors a fee for the printing of their color figures. Please contact our offices for exact pricing and details.

As soon as your article is published, you will receive an automated email with your shareable link.

If you have not already done so, we strongly recommend that you upload the step-by-step protocols used in this manuscript to protocols.io (https://protocols.io), an open online resource that allows researchers to share their detailed experimental know-how. All uploaded protocols are made freely available and are assigned DOIs for ease of citation. Protocols and Nature Portfolio journal papers in which they are used can be linked to one another, and this link is clearly and prominently visible in the online versions of both. Authors who performed the specific experiments can act as primary authors for the Protocol as they will be best placed to share the methodology details, but the Corresponding Author of the present research paper should be included as one of the authors. By uploading your Protocols onto protocols.io, you are enabling researchers to more readily reproduce or adapt the methodology you use, as well as increasing the visibility of your protocols and papers. You can also establish a dedicated workspace to collect your lab Protocols. Further information can be found at https://www.protocols.io/help/publish-articles.

You can use a single sign-on for all your accounts, view the status of all your manuscript submissions and reviews, access usage statistics for your published articles and download a record of your refereeing activity for the Nature Portfolio.

Nature Cell Biology encourages authors presenting evidence for cell, biological, molecular, and genetic interactions to consider communicating these findings using Biofactoid (https://biofactoid.org/). This tool helps users share a searchable representation of interactions (e.g. binding, gene expression, post-translational modification) between genes, gene products, or chemicals. Information added to Biofactoid, with author attribution, is shared on social media and public databases, such as Pathway Commons, where it can be discovered and analyzed in the context of a large and growing corpus of knowledge.

Please feel free to contact us if you have any questions.

With kind regards,
Daryl

Daryl Jason Verzosa David, PhD

Senior Editor, Nature Cell Biology
Advisory Editor, npj Biological Physics and Mechanics
Nature Portfolio

Heidelberger Platz 3, 14197 Berlin, Germany
Email: daryl.david@nature.com
ORCID: https://orcid.org/0000-0002-9253-4805

Click here if you would like to recommend Nature Cell Biology to your librarian
http://www.nature.com/subscriptions/recommend.html#forms

** Visit the Springer Nature Editorial and Publishing website at www.springernature.com/editorial-and-publishing-jobs for more information about our career opportunities. If you have any questions please click here.**

Open Access This Peer Review File is licensed under a Creative Commons Attribution 4.0 International License, which permits use, sharing, adaptation, distribution and reproduction in any medium or format, as long as you give appropriate credit to the original author(s) and the source, provide a link to the Creative Commons license, and indicate if changes were made.
In cases where reviewers are anonymous, credit should be given to 'Anonymous Referee' and the source.
The images or other third party material in this Peer Review File are included in the article's Creative Commons license, unless indicated otherwise in a credit line to the material. If material is not included in the article's Creative Commons license and your intended use is not permitted by statutory regulation or exceeds the permitted use, you will need to obtain permission directly from the copyright holder.
To view a copy of this license, visit https://creativecommons.org/licenses/by/4.0/

Rebuttal letter NCB-A57858A

All the new text has been marked in **blue**.

Reviewer #1 (Remarks to the Author):

The reviewer: *A. Summary of the key results:*
The abstract accurately summarizes the paper

B. Originality and significance:
The study is novel and interesting. There has been a lot of interest in spindle scaling, but published studies focused on reduction divisions in early embryos. This paper focuses on a more general problem, scaling of somatic cell spindles across species. This allows the authors to investigate a large range of genome sizes. The study encompasses plants and microorganisms, which is very nice from a significance perspective.

The authors: We thank the reviewer for appreciating the novelty and significance of the study. In response to Reviewer #1, we performed additional experiments and analyses to explicitly test assumptions about chromatin density, metaphase plate geometry, and chromosome crowding:

- Measured metaphase plate thickness across diverse eukaryotic species and combined these measurements with plate width to estimate metaphase plate volume and effective chromatin density across species.
- Calculated effective metaphase plate chromatin density and demonstrated that it is approximately constant across genome sizes, indicating near-linear scaling between plate volume and genome size.
- Measured changes in metaphase plate width, thickness, spindle width, and chromatin density following acute reactivation of CENP-E, allowing direct assessment of how progressive chromosome entry into the plate affects plate dimensions and density.
- Added analyses of metaphase plate aspect ratio (width/thickness) to assess conservation of plate shape across species.

Together, these new results directly address concerns about constant-density assumptions and demonstrate that chromosome crowding primarily drives metaphase plate widening while chromatin density and plate shape remain largely conserved.

The reviewer: *C. Data & methodology: validity of approach, quality of data, quality of presentation:*

Overall good, but I feel the paper could lay out the conceptual arguments more clearly for a general audience, esp. early in the results. Finding that a linear dimension of an object scales as the cube root of its mass is the expected result if the object keeps a constant shape as its mass changes. The authors find that metaphase width indeed increases as the cube root of mass, but spindle width does not. This means that the shape and/or density are not constant. Figure 1 should include an estimate of density based on length and width and perhaps a shape parameter also.

The authors: We thank the reviewer for highlighting the importance of explicitly testing the constant-density assumption. First, we note that spindle width follows a power-law scaling with genome size similar to that of metaphase plate width, though with a weaker correlation (Fig. 1 and Extended Data Fig. 1). The higher scaling exponent for spindle width (~0.41) could be due to the contribution of rod-like spindles that characterize some species with small genomes, such as yeasts. Second, following the reviewer's suggestion, we measured metaphase plate thickness across eukaryotes and estimated both the plate volume and the average density of chromatin in the metaphase plate from the genome size and the plate volume $V = \pi \cdot \left(\frac{W}{2}\right)^2 \cdot T$, where W and T are measured metaphase plate width and thickness, respectively (Extended Data Table 1). New results have been incorporated into text and new Figures 1 and Extended data Fig. 1.

"Previous analyses of condensed metaphase chromosomes revealed a remarkably uniform chromatin density across human chromosomes and a subset of eukaryotic species (~90 Mbp μm^{-3})[27–29]. To assess whether this constancy extends to the scale of the entire metaphase plate, we estimated plate volume from measured width and thickness across diverse eukaryotes (Methods). Plate volume scaled with genome size according to a power law with an exponent of 0.96 ($R^2 = 0.91$, $p = 10^{-13}$; Fig. 1e), indicating a near-linear increase in plate volume

with chromatin amount. Accordingly, effective metaphase plate density (6.27±0.99 Mbp μm^{-3}) was roughly constant across genome sizes (Extended Data Fig. 1e). The lower effective density of the whole metaphase plates relative to isolated chromosomes[27–29] likely reflects inclusion of inter-chromosomal spaces in plate volume estimates. Plate shape was likewise similar across eukaryotes, with a width to thickness aspect ratio of 1.89 ± 0.59 (Extended Data Fig. 1f, g). Together, these results indicate that metaphase plate density and shape are conserved across species.*"*

The reviewer: *D. Appropriate use of statistics and treatment of uncertainties:*
OK

E. Conclusions: robustness, validity, reliability: Strong, though as detailed above the authors should include a specific estimate of DNA density in the metaphase plate, inferred from width and length, and use that to both explain why a mechanical model is needed and how it solves the non-constant density part of the problem.

The authors: We addressed this comment both experimentally and theoretically. We measured chromatin density across eukaryotes, as described in the response to point C. Additionally, we have measured chromatin density within the plate in RPE1 cells of different ploidy, as well as after acute reactivation of CENP-E. These results are now included as Extended Data Figure 2c and Figure 2f, g and Extended Data Fig. 5a-c, and the corresponding text has been added to the manuscript:

"In polyploid cells, we reasoned that the higher chromosome number would increase chromatin density within the metaphase plate, reflecting chromosome crowding and the resulting inter-chromosome pushing forces. Indeed, chromatin density increased after ploidy doubling by 19% in tetraploid and 17% in hypooctaploid RPE1 cells (Extended Data Fig. 3e). In parallel, metaphase plate width and thickness increased by 19% and 28% in tetraploids, and by 29% and 37% in hypooctaploids (Fig. 2b, Extended data Fig. 2f). The overall increase over all ploidies was higher for the plate dimensions than for chromatin density. These data indicate that while higher ploidy modestly elevates chromatin density, cells primarily accommodate additional chromatin through expansion of the metaphase plate volume."

"Based on the chromosome crowding hypothesis, we predicted that metaphase plate width and chromatin density would increase as additional chromosomes entered the plate following acute reactivation of CENP-E[49]. Indeed, as chromosomes progressively accumulated within the metaphase plate, both plate width and chromatin density increased, accompanied by an increase in spindle width and a decrease in plate thickness (Fig. 2g, Extended Data Fig. 5a-c, Extended Data Movie 3). Similar trends were observed in fixed CENP-E-depleted cells (Extended Data Fig. 5d, e) and in published images of near-haploid human tumor HAP1 cells[50], which displayed metaphase plates that were 18% narrower than those of near-diploid cells (Extended Data Table 2). Together, these observations demonstrate that inter-chromosome pushing forces, reflected by changes in metaphase plate width and chromatin density, increase as the number of chromosomes within the plate rises."

In the model, we addressed the non-constant density by exploring the idea that the relationship between genome size and chromosome volume may be nonlinear. In this case, we found a disagreement between the scaling in the model and the one observed across eukaryotes, which additionally supports our basic assumptions in the model:

"The scaling predicted by the model is robust to changes in the number and physical properties of microtubules and chromosomes (Extended Data Fig. 8e; Methods). Moreover, the scaling does not depend on chromosome shape: incorporating a more realistic prolate spheroidal chromosome geometry produces similar scaling behavior (Extended Data Fig. 8e; Methods). In contrast, the scaling directly depends on the relationship between genome size and chromosome volume. When this relationship is nonlinear, the scaling exponent changes markedly (Extended Data Fig. 8f). The model therefore suggests that the experimentally observed scaling exponent of 0.33 arises from a linear relationship between chromosome volume and genome size."

The reviewer: *F. Suggested improvements: experiments, data for possible revision:*
As detailed above. No new experiments are needed. This is a very interesting paper. The data are very simple. But I feel the conceptual layout of the paper can be improved.

G. References: appropriate credit to previous work?:
OK

H. Clarity and context: lucidity of abstract/summary, appropriateness of abstract, introduction and conclusions:
OK, but see comments on conceptual arguments as detailed above.

The authors: We worked on laying out conceptual arguments better, as detailed in the previous comments.

Reviewer #2 (Remarks to the Author):

The reviewer: *Accurate chromosome segregation during mitosis requires the precise organization of chromosomes within the spindle apparatus. While previous studies have examined how spindle size scales with cell and nuclear dimensions, the influence of genome size and chromosome number on spindle architecture remains less well understood.*

This study presents a universal scaling law that links metaphase plate width and spindle width to genome size and chromosome number across diverse eukaryotic systems. Using a physical model and compression experiments on mitotic cells, the authors elucidate how inter-chromosomal pushing forces shape spindle morphology. They propose that chromosome crowding plays a central role in determining metaphase plate width/spindle width, whereas spindle length is likely governed by independent mechanisms, such as the activity of microtubule-associated proteins. These findings deepen our understanding of the biophysical principles that govern cell division in eukaryotes, and they are of interest to NCB readers. However, several critical issues must be addressed before the manuscript is suitable for publication.

The authors: We are grateful to the reviewer for a comprehensive assessment of the manuscript and for providing positive and constructive comments. In response to Reviewer #2, we performed an extensive set of new experiments and substantially expanded quantitative analyses to test the roles of ploidy, chromosome number, chromatin amount, cytoplasmic forces, and microtubules, as well as extended the theoretical model:

- Developed an improved polyploidization protocol (thymidine synchronization, TP53 depletion, cytokinesis failure induction, extended recovery, and metaphase enrichment) to obtain higher and more reproducible ploidy states.
- Measured metaphase plate width, thickness, spindle width, spindle length, and cell area across multiple ploidy levels using the improved protocol.
- Quantified total DNA intensity and effective chromatin density within metaphase plates across ploidy levels.
- Performed acute reactivation of CENP-E after chemical inhibition to examine how progressive chromosome entry affects metaphase plate width, thickness, spindle width, and chromatin density.
- Expended experiments on mitosis with an unreplicated genome (MUG) and measured spindle dimensions and cell size to test effects of reduced chromatin amount on the spindle and altered chromosome structure.
- Performed acute nocodazole treatments on metaphase spindles to test the contribution of microtubules to maintaining spindle and plate width.
- Performed acute hyperosmotic and hypoosmotic shocks to test the role of cytoplasmic crowding forces on spindle and metaphase plate dimensions.
- Clarified analyses of patient-derived organoids and cell selection criteria and ploidy proxies.
- Extended the theoretical description of chromosome shape by considering a more realistic prolate spheroid geometry.

Together, these new experiments and analyses demonstrate that metaphase plate width and spindle width are primarily determined by chromatin amount and chromosome crowding, while microtubules and cytoplasmic forces make secondary contributions.

The reviewer: *Major remarks:*

1. *In Box 1, the formula F=−ch3/2 likely derives from the Hertz contact model. The exponent 3/2 is critical for their model and may dominate the resulting power law exponent. The authors should clarify why this model was chosen, how biologically relevant it is (including assumptions), and whether alternative models were tested or considered.*

The authors: We thank the reviewer for this comment. To answer this question, we explored more broadly what assumptions in the model are critical for the resulting power law exponent. We found that the exponent depends predominantly on the relationship between chromosome volume and genome size, whereas the elastic properties included in the Hertz model have a minor effect. In addition, we explored an alternative model where chromosome geometry was described as a prolate spheroid, which also had a minor effect. These new results are now described in the main text:

“The scaling predicted by the model is robust to changes in the number and physical properties of microtubules and chromosomes (Extended Data Fig. 8e; Methods). Moreover, the scaling does not depend on chromosome shape: incorporating a more realistic prolate spheroidal chromosome geometry produces similar scaling behavior (Extended Data Fig. 8e; Methods). In contrast, the scaling directly depends on the relationship between genome size and chromosome volume. When this relationship is nonlinear, the scaling exponent changes markedly (Extended Data Fig. 8f). The model therefore suggests that the experimentally observed scaling exponent of 0.33 arises from a linear relationship between chromosome volume and genome size.”

In addition, we clarified the choice and relevance of the model’s assumption in Methods:

“This description of inter-chromosome pushing forces considers elastic properties of homogenous spheres and is valid for small deformations, which is the case in our system (Fig. 3a, d). We do not consider short-range electrostatic surface interactions because previous experiments have shown that they have a small effect on the metaphase plate width[36].”

The reviewer: 2. *In Fig. 1, the authors show that metaphase plate width correlates with genome size, but not with chromosome number. How do they explain this? This referee would like to see how plate width scales with genome size when chromosome number is held constant, and conversely, how it scales with chromosome number when genome size is held constant, across different eukaryotes.*

The authors: We thank the referee for this question. To explore the dependence on the chromosome number, we compared experimental and theoretical results in Fig. 3c. For experimental data, we took 4 species with a similar genome size but a very different chromosome number, and found that the metaphase plate width was roughly constant, 14-16 μm, whereas the chromosome number spanned a large range from 6 to 46. We have added these new findings in Results:

„ To test the case of constant total volume of chromosomes, we compared 4 species that have a similar genome size (5–8 Gbp), but a substantially different chromosome number (6–46, Extended Data Table 1). These species have a similar metaphase plate width, in agreement with a roughly constant width predicted by the model (Fig. 3c). Thus, irrespective of the way the chromosome number was altered experimentally, and even when comparing different species, the experimental data agree with the model’s prediction that the spindle shape is determined by the total chromosome volume.”

We also explored how plate width scales with genome size when chromosome number is held constant:

“To explore the role of chromosome number in the observed scaling, we examined the scaling behavior both across varying chromosome numbers and when chromosome number was held approximately constant. Across all species, metaphase plate and spindle dimensions showed no correlation with chromosome number, in contrast to their strong correlation with genome size (Fig. 1f, and Extended Data Fig. 1h-j). Focusing on a subset of species with similar chromosome numbers, ranging from 32 in yeast to 42 in wheat (Extended Data Table 1), we found that metaphase plate width remained strongly correlated with genome size despite the narrow range of chromosome numbers ($R^2 = 0.97$, $p = 0.0028$). We infer that the total amount of chromatin in the metaphase plate, rather than chromosome number, underlies the observed scaling.”

The reviewer: *3. In Fig. 2, it’s unclear why the authors switch from genome size to interphase nuclear area. How do the authors use the interphase nuclear area as a proxy for chromosome number? In Extended Data Fig. 4d, nuclear area does not increase with chromosome number, which contradicts their claim. This reviewer agrees that nuclear area generally reflects ploidy numbers, making it a useful proxy for estimating ploidy. However, the relationship is not perfectly fixed and can be influenced by cell type and chromatin state. Nuclear volume is a more precise metric than nuclear area for this purpose. Since the nucleus has a 3D shape, its total volume—not just area—better reflects chromosome number.*

The authors: We thank the reviewer for this comment. We understand that the interphase nuclear area is not a direct measurement of ploidy, but it serves as a good proxy, in accordance with previous literature (Gemble, S. et al., 2022.). We agree that the data presented in Extended Data Fig. 4d did not show an increase in nuclear area upon acute polyploidization. However, the data presented in Fig. 2c from live-imaged cells, which avoids

issues of inefficient polyploidization by tracking each cell from prophase to metaphase, clearly showed that nuclear area increases with ploidy. To improve the efficiency of polyploidization, which we suspect affected the lower nuclear areas due to an overrepresentation of cells with lower ploidy in fixed cells, we developed a new polyploidization protocol. Specifically, we replaced palbociclib G1 synchronization with thymidine, which in our hands resulted in a more efficient release after synchronization. We also depleted TP53 prior to synchronization in RPT3 clones to further enhance the release from synchronization and the subsequent division of polyploid cells. In addition, we replaced the induction of mitotic slippage with cytokinesis failure. After polyploidization, we extended the incubation period after inducing polyploidization compared to the previous protocol to increase the likelihood of observing the next division of polyploid cells, which are known to have a delayed cell cycle compared to diploid cells (Gemble, S. et al., 2022.). Finally, we used a proteasome inhibitor to enrich cells in metaphase. Please see Methods and Extended data Fig. 3d for details.

Using this improved protocol, we analyzed the cell area and spindle and metaphase plate parameters on a cell-by-cell basis and observed a clear increase in cell area with ploidy (new Extended Data Fig. 3f), similar to the prophase nuclear area in live-imaged cells (now Figure 2c). For this reason, we have removed Extended Data Fig. 4d. We have incorporated new findings into Results:

"To test the relationship between the metaphase plate width and ploidy on a cell-by-cell basis, we used the nuclear area in prophase as an established proxy for the ploidy in each cell[41]. The metaphase plate width in live-imaged cells correlated with the nuclear area and a power-law fit yielded an exponent of 0.29 (Fig. 2c), matching the scaling found across eukaryotes (Fig. 1d). In fixed cells, metaphase cell area was used as an indirect proxy for ploidy because it increased markedly with ploidy (Extended Data Fig. 3f). Consistent with this, metaphase plate width showed a similar power-law scaling with cell area as with nuclear area (Fig. 2c, Extended Data Fig. 3g), indicating that the scaling relationship is robust to the choice of ploidy proxy."

The reviewer: *4. For organoids, how were the target cells selected? Could the nucleus or spindle be compressed by neighboring cells in PDOs, as shown in Fig. 4? The authors also need to explain what the "24Ta, 24Tb, 9T…" cell types refer to, and how they define lower or higher ploidy. For panels e and c, consider using log-log plots.*

The authors: We appreciate this comment. In organoids, we quantified all cells entering metaphase, excluding highly tilted cells, as now specified in the Methods. In PDOs we did not observe the aberrant metaphase-plate morphology typical of spindle compression (Figure 4). Consistently, mitotic duration in tumor PDOs was unchanged relative to control PDOs (data not shown) and matched that of non-transformed human cell lines (Yang et al., 2008). This contrasts with compressed cells, which show prolonged mitosis (Extended Data Fig. 6i).

Methods: "In PDOs, we measured all cells that entered metaphase, excluding only those that were highly tilted."

Regarding the labeling, we have now defined all terms in the corresponding figure caption. Additionally, we replaced the terms *higher* and *lower ploidy* with *larger* and *smaller nuclear area*.

Figure 2 caption: "**c**, **e**, Metaphase plate width, W, versus interphase nuclear area, A, in cells of different ploidies (**c**), and in colorectal cancer (CRC) patient-derived organoids (PDOs) lines (**e**), each obtained from independent patient tumor samples (24Ta, 24Tb, 9T, T7, P27T, where "T" indicates tumor) and control sample (P1N, where "N" indicates normal)[47], with a power-law fit ($W \propto A^{0.29}$, $R^2 = 0.57, p = 2.57 \times 10^{-31}$, and $W \propto A^{0.41}$, $R^2 = 0.63, p = 4.6 \times 10^{-25}$, respectively), and 95% confidence intervals. In (**c**), orange dots represent RPT3 and yellow RPT1 cells. Number of cells: 58, 28, 36, 51, 7 (c); 58, 59, 21, 27, 23 (e). **d,** CRC PDOs expressing H2B-GFP (top), with cells of smaller (1) and larger (2) ploidy enlarged (bottom). Movies obtained from[47]."

Due to the limited variability in the interphase nuclear area (less than an order of magnitude), we have retained the linear-linear plots.

The reviewer: *5. In Fig. 2, another way to reflect pushing forces between chromosomes at the metaphase plate is by measuring their DNA intensity. According to the authors' model, higher crowding and pushing forces would suggest greater average intensity. The authors should measure DNA intensity across different ploidy levels, MUG cells, and compression conditions.*

The authors: We thank the reviewer for this excellent suggestion. To address it, we have quantified both the total chromatin signal by DAPI staining and calculated effective chromatin density within the metaphase plate across multiple ploidy levels in a newly acquired experimental dataset (see response to comment 3) with DNA staining kept constant across different ploidy levels (Methods). These results are now included in Extended Data Fig. 2e and Extended Data Fig. 3e and described in the text.

"Total chromosome signal intensity increased nearly twofold upon ploidy doubling (Extended Data Fig. 2e), confirming successful polyploidization."

"In polyploid cells, we reasoned that the higher chromosome number would increase chromatin density within the metaphase plate, reflecting chromosome crowding and the resulting inter-chromosome pushing forces. Indeed, chromatin density increased after ploidy doubling by 19% in tetraploid and 17% in hypooctaploid RPE1 cells (Extended Data Fig. 3e). In parallel, metaphase plate width and thickness increased by 19% and 28% in tetraploids, and by 29% and 37% in hypooctaploids (Fig. 2b, Extended data Fig. 2f). The overall increase over all ploidies was higher for the plate dimensions than for chromatin density. These data indicate that while higher ploidy modestly elevates chromatin density, cells primarily accommodate additional chromatin through expansion of the metaphase plate volume."

Given the experimental complexity of the MUG and compression assays, we did not extend this analysis to those conditions. However, we have tested if the density of chromatin in the metaphase plate acutely changes in live-imaged cells as more chromosomes enter the plate by performing acute reactivation of CENP-E activity after chemical inhibition. These new results have been incorporated into the text:

"Based on the chromosome crowding hypothesis, we predicted that metaphase plate width and chromatin density would increase as additional chromosomes entered the plate following acute reactivation of CENP-E[49]. Indeed, as chromosomes progressively accumulated within the metaphase plate, both plate width and chromatin density increased, accompanied by an increase in spindle width and a decrease in plate thickness (Fig. 2g, Extended Data Fig. 5a-c, Extended Data Movie 3). Similar trends were observed in fixed CENP-E-depleted cells (Extended Data Fig. 5d, e) and in published images of near-haploid human tumor HAP1 cells[50], which displayed metaphase plates that were 18% narrower than those of near-diploid cells (Extended Data Table 2). Together, these observations demonstrate that inter-chromosome pushing forces, reflected by changes in metaphase plate width and chromatin density, increase as the number of chromosomes within the plate rises."

Differences between acute increase of chromosome number and polyploidization experiments are discussed:

"Metaphase plate thickness, reflecting chromosome alignment, also changed upon polyploidization. We speculate that this arises from altered resources available per chromosome for attachment to spindle microtubules, including kinetochore proteins and MAPs regulating microtubule nucleation and dynamics. Our transcriptome analysis indicates that the mitotic transcriptome increases sublinearly with ploidy[46], implying fewer mitotic factors per chromosome. These changes may weaken microtubule attachments, enhance kinetochore oscillations, and overall impair chromosome alignment following polyploidization."

The reviewer: *6. Since spindle size has been shown to scale with cell size, have the authors measured cell size across different ploidy levels? This might explain why spindle length remains unchanged in MUG cells.*

The authors: We thank the reviewer for this question. We have now measured cell size across different ploidy levels obtained using the improved polyploidization protocol and observed that cell size increases sublinearly with ploidy (see comment 3 and 7 for details). In MUG cells, we measured cell size in both MUG and control cells. These observations are now incorporated into the text and Extended Data Fig. 3f and Extended Data Fig. 5f, g.

"In fixed cells, metaphase cell area was used as an indirect proxy for ploidy because it increased markedly with ploidy (Extended Data Fig. 3f). Consistent with this, metaphase plate width showed a similar power-law scaling with cell area as with nuclear area (Fig. 2c, Extended Data Fig. 3g), indicating that the scaling relationship is robust to the choice of ploidy proxy."

"Fourth, we reduced the amount of chromatin in the metaphase plate and disrupted chromosome structure by inducing mitosis with an unreplicated genome (MUG)[51] in the U2OS osteosarcoma cell line (Fig. 2h). In MUG cells, where centromeres are aligned on the spindle but most of the uncondensed chromatin is displaced in the cytoplasm, spindles were 31% thinner without changes in their length or cell size (Fig. 2i; Extended data Fig. 5d, e; note that the metaphase plate width cannot be determined due to uncondensed chromatin). These experiments indicate that the amount of chromatin and its mechanical properties determine spindle width."

The reviewer: *7. In Fig. 2, metaphase plate thickness appears to vary with ploidy or chromosome number. However, why is there no difference in metaphase plate width/thickness between hypotetraploid cells and tetraploid cells, despite genome density being different? Can the authors comment on that?*

The authors: We agree with the reviewer. A potential explanation, which we omitted from the manuscript due to its speculative nature, is that our hypotetraploid cells are adapted clones (RPT3, RPT1, HPT1, and HPT2), whereas the tetraploid cells were acutely generated from RPE1 or HCT116 lines. Thus, we do not directly compare hypotetraploid and tetraploid cells, but rather cells of different ploidy levels derived from the same genetic background.

The reviewer: *8. In Figs. 2h/i and Extended Figs. 4f/g, Cenp-E–depleted chromosomes on the metaphase plate appear more dispersed. However, based on their model, they should be more compacted due to their reduced size or stiffness. How do the authors explain this discrepancy? Also, spindle lengths in these cells appear longer—could this be an adaptation to maintain global force balance? If so, does this imply that spindle length is indirectly affected by chromosome number?*

The authors: We agree with the reviewer and acknowledge this as a limitation of using CENP-E depletion. These limitations and the underlying reasons are now noted in the Methods.

Methods: "CENP-E depletion activates the spindle assembly checkpoint and prolongs mitosis, causing increased microtubule pulling and chromosome dispersion within the metaphase plate[49]. Additionally, the extent of metaphase oscillations decreases as cells progress closer to anaphase[108], producing effectively higher plate thickness in prometaphase cells. The longer spindle length in cells with more unaligned chromosomes likely reflects a prometaphase-like state[49], consistent with previous reports that spindle length decreases from prometaphase to metaphase[69]."

Additionally, we have tested if the density of chromosomes changes as more chromosomes enter the plate by performing acute reactivation of CENP-E activity after chemical inhibition. We have discussed those findings in response to comment 5. Of note, we see there that metaphase plate thickness decreases following reactivation of CENP-E activity.

The reviewer: 9. *In Extended Fig. 3, the authors report a correlation between plate thickness and interphase nuclear area in both RPE1 and PDO cells. Since metaphase plate volume is proportional to $W^2 \cdot T$ (where W is width and T is thickness), both parameters should be included in the model. Accordingly, the authors should complete the metaphase thickness dataset and consider updating their model—perhaps using a multilayer elastic-ball approach—to incorporate thickness.*

The authors: We thank the reviewer for this interesting observation and suggestion. Even though we did not directly include thickness in the model, we explored under which conditions the thickness increases. By performing the stability analysis of the spindle, we find that thickness increases for higher ploidies. This new theoretical analysis has been incorporated into Results:

„ To test under what conditions the inter-chromosome pushing forces are large enough to displace the chromosomes from the metaphase plate, we performed stability analysis of the metaphase plate in the extended model where chromosomes are aligned at the spindle midplane by an elastic spring (Methods). We obtain that

chromosome alignment at the metaphase plate is stable if the spring is stiff enough, which is the case for diploid human cells (Extended Data Fig. 2f-i). In contrast, in cells with higher ploidies, the metaphase plate can become unstable for the same spring stiffness, in agreement with the experimentally increased thickness of the metaphase plate (Extended Data Fig. 2f-i)."

We have also measured metaphase plate thickness across eukaryotes. We found that metaphase plate thickness scales with genome size following a power law with an exponent of 0.29 (Extended Data Fig. 1d), but the correlation is weaker compared to metaphase plate width. These findings have been incorporated into the Results section.

"Spindle length and width, as well as metaphase plate thickness, also show power-law scaling with genome size (Extended Data Fig. 1b-d). However, their correlation with genome size is weaker compared to that of metaphase plate width (z-score = 2.76, p = 0.0029, z-score = 2.13, p = 0.017, and z-score = 4.40, p = $5x10^{-5}$ for spindle length, width, and metaphase plate thickness, respectively). Together, these relationships identify metaphase plate width as the key spindle parameter scaling with genome size."

We have also completed the metaphase plate thickness dataset by measuring RPE1 cells of different ploidy after cytokinesis failure (see comment 3). In RPE1 cells, metaphase plate thickness increased with ploidy, but not consistently across various polyploidization protocols and measurements schemes (Extended Data Fig. 2). We acknowledge this in Results:

"We found that metaphase plate width increased with ploidy across all cell types and polyploidy-induction methods, whereas metaphase plate thickness, spindle length, and spindle width increased in most, but not all, cases (Fig. 2b, Extended Data Fig. 2f-i, and Movie 1). The spindle or metaphase plate widening following polyploidization was not dependent on the presence of centrioles at the spindle poles in RPE1 cells (Extended Data Fig. 3a-c), as well as in naturally acentriolar mouse embryonic cells[43] (Extended Data Fig. 3d, Table 2). Together, these results demonstrate that the metaphase plate width scales robustly with chromatin content upon polyploidization."

The reviewer: 10. *In Fig. 3, the authors state: "We use spheres as the simplest geometric representation of chromosome shape, taking their volume as a proxy for DNA content." The authors should clarify why and how they chose elastic spheres, referencing studies 51–53. Since they use DNA content in their calculations, they clearly need to define this term along with their assumptions (see also your first minor comment).*

The authors: We revised the description of the model to clarify the choice of elastic spheres. We also replaced the expression "DNA content" with a more precise term "chromatin amount":

„To directly test the chromosome crowding hypothesis and estimate the resulting inter-chromosome pushing forces, with the ultimate aim of explaining the spindle scaling law, we introduced a physical model of the spindle. We chose a simple approach where the spindle is composed of chromosomes and microtubules, while omitting proteins unrelated to the chromosome crowding hypothesis, such as microtubule-associated proteins (MAPs). We model each chromosome as a sphere because this is the simplest geometric object characterized solely by its radius, and we use the sphere volume as a proxy for the amount of chromatin (Fig. 3a, and Box 1). In our model, the chromosomes are compressible in response to forces, based on previous measurements of chromosome elasticity[59]. They are attached to microtubules and positioned in the spindle midplane, representing the metaphase plate. The microtubules, which extend between the spindle poles, push the chromosomes inward towards the spindle axis through their bending forces (Fig. 3a). In response, chromosomes exert inter-chromosome pushing forces as they squeeze against one another. Together, this balance of microtubule bending forces and inter-chromosome pushing forces defines the mechanical equilibrium that sets the chromosome positions and thereby the width of the metaphase plate and the overall spindle architecture (Box 1, Methods)."

The reviewer: 11. *In Figs. 3b/c, the authors infer that the scaling exponent between W and V is 1/3, and between W and N is 1/2. However, these conclusions are incomplete without accounting for plate thickness, as noted in comment 9.*

The authors: We extended our calculation on the scaling between *W* and *N*, although we did not include thickness. Interestingly, we found that *W* does not scale with *N* when the total volume of chromosomes is constant, which provides further support to our main hypothesis:

"We next alter the chromosome number while keeping constant either the volume of individual chromosomes or their total volume. In the case of constant volume of individual chromosomes, where an increased number of chromosomes corresponds to the increased total amount of chromatin, the model predicts a power-law scaling of the metaphase plate with an exponent of 0.43 (Fig. 3b). In contrast, in the case of constant total volume of chromosomes, where individual chromosomes become smaller as their number increases, the model predicts a roughly constant metaphase plate width except for narrower plates at small chromosome numbers (Fig. 3c). Thus, spindle shape is set primarily by the total chromosome volume rather than by their number."

The reviewer: *12. In their model, have the authors considered the role of cytoplasmic forces that could push mitotic chromosomes via crowding? Osmotic shock experiments could help test this. Additionally, could microtubules themselves contribute to chromosome pushing? Please clarify.*

The authors: We are especially grateful to the reviewer for highlighting the possible role of cytoplasmic forces and microtubules in regulating chromosome crowding. Following this suggestion, we performed new experiments inducing acute osmotic shock, as well as acute nocodazole shock, on live-imaged metaphase spindles. We have incorporated these new data into the revised Figure 2 as well as new Figure 5, together with the data originally presented in Figure 4i–k. As our experiments showed that cytoplasmic forces on chromosomes in unperturbed cells are relatively modest, we did not extend the model to include these forces. The new experimental findings are described in Results:

"To test the alternative hypothesis that spindle width is maintained by microtubule-generated forces[25,37], we acutely depolymerized microtubules in the metaphase spindle in diploid RPE1 cells using 1 μM nocodazole (Fig. 2j, Extended Data Fig. 6a, Extended Data Movie 4). Nocodazole caused a rapid and pronounced reduction in spindle length (65%), whereas spindle width and metaphase plate width remained unchanged over 7 minutes, and control metaphase cells showed no changes (Fig. 2k, Extended Data Fig. 6b, c). Immunofluorescence confirmed near-complete microtubule depolymerization within ~7 minutes of nocodazole treatment (Extended Data Fig. 6d, e). These observations show that metaphase spindle width is maintained primarily by the physical properties of metaphase chromatin and inter-chromosome pushing forces."

"To complement our results on external compression forces, we asked whether endogenous forces, such as cytoplasmic crowding[70], contribute to maintaining spindle and metaphase plate dimensions. We applied hyperosmotic and hypoosmotic solutions to RPE1 cells in metaphase, which have been shown to increase or decrease cytoplasmic density by approximately 45%, respectively[71] (Fig. 5a, Extended Data Movie 4). Hyperosmotic shock caused an immediate narrowing of the spindle (~14%) and the metaphase plate (~18%), followed by gradual but extensive spindle elongation (~60%, Fig. 5a, b, Extended Data Fig. 6d, g). These results indicate that increased cytoplasmic forces acutely compress the metaphase plate. In contrast, hypoosmotic shock had no immediate effect but led to gradual shortening of the spindle and widening of both the spindle and metaphase plate (Fig. 5a, b, Extended Data Fig. 6d, g). This finding shows that decreased cytoplasmic density triggers slower remodeling of the spindle and metaphase plate, implying that cytoplasmic pushing forces on chromosomes in unperturbed cells are relatively modest."

The reviewer: 13. *In Fig. 4, this reviewer is not fully convinced by their compression experiments. While well-performed, they do not clearly address their central hypothesis. After compression along the Y-axis, it is unclear whether inter-chromosome pushing forces along the Z-axis increase. Therefore, concluding that Wz expands due to inter-chromosome forces may not be justified. Moreover, even if chromosomes are not in contact on the spindle (e.g., only the two outer ones are), the plate width can still expand upon compression due to the elastic properties of microtubules, which behave like springs. A different or more direct experiment would better support the model.*

The authors: We thank the reviewer for this point. As we cannot measure inter-chromosome forces experimentally, this is the point where the model helps to fill the gap. Our model suggests that, after compression along the Y-axis, inter-chromosome pushing forces along the Z-axis increase. Based on the quantitative agreement between the model and the experimentally perturbed spindle shape, we conclude that

the metaphase plate indeed expands due to inter-chromosome pushing forces. To visualize this theoretical result more clearly, we have replotted Figure 4b on a log-log scale. In addition, we explored the role of the elastic properties of microtubules in the model. When the flexural rigidity of microtubules was reduced 10 times, we found only minor change in the metaphase plate width across species (Extended Data Fig. 8e), indicating that microtubule elasticity plays a minor role.

We indirectly tested the role of microtubules by compressing the spindles of MUG cells, characterized by decondensed and largely displaced chromatin. In these cells, microtubules presumably contribute more to the mechanical response than in control cells. However, we observed that spindle widening was less pronounced than in controls, suggesting that chromosomes, rather than microtubules, are the primary determinants of the spindle's response to compression.

"Spindles in MUG cells also widened upon compression, but only by ~23% (Fig. 4e, g), whereas spindle length remained unchanged (Extended data Fig. 7f, and Extended Data Movie 2). Both experiments agree with the model's predictions that the spindle widens under compression (compare Fig. 4c, d with Fig. 4g, h). For a quantitative comparison, we identified a compression coefficient of 100 pN/µm, where the decrease in spindle height predicted by the model is similar to that observed in experiments. The model suggests that the inter-chromosome pushing forces increased 3-fold in the compression experiments (Fig. 4b). As in experiments, the model predicts a larger spindle widening for the parameters corresponding to control than to MUG cells, 39% and 34%, respectively (Fig. 4a, c). Thus, the biomechanical tests inspired and interpreted by the model confirmed the chromosome crowding hypothesis."

In addition, two lines of evidence argue against microtubule dynamics as the primary driver of spindle widening and instead support a role for chromosome crowding and microtubule pivoting. First, Dumont and Mitchison (2009) showed that spindles widen but do not lengthen under compression when microtubule dynamics are suppressed with taxol. Second, our nocodazole experiments acutely depolymerized microtubules, producing a rapid loss of microtubules and reduction of spindle length with little change in spindle or metaphase-plate width (see response to comment 12 for details). Together, these observations indicate that microtubule dynamics is not required for maintaining spindle width and are consistent with inter-chromosomal pushing forces, chromatin incompressibility and mechanical pivoting of microtubules as the main contributors to width changes. This is now acknowledged in the Discussion:

"Compression-induced spindle widening occurs even in taxol-treated spindles, suggesting it is independent of microtubule dynamics[66]."

The reviewer: 14. *In Fig. 5, they claim that when DNA length increases by a factor of n, chromosome volume increases n-fold—this holds only if DNA density remains constant. Similarly, chromosome length increases by n^1/3 only if the shape remains constant (which is probably not the case in reality). The same logic applies to metaphase plate width: it increases with n^1/3 only if plate thickness scales in the same way. The authors should state all their assumptions clearly and explain how they diverge from biological reality.*

The authors: We thank the reviewer for highlighting these assumptions. The assumption that when DNA length increases by a factor of n, chromosome volume increases n-fold was shown in the literature, suggesting that DNA density remains constant across vertebrates and flowering plants (Kramer et al., 2021). We have now explicitly addressed chromatin density in the metaphase plate and its variability across species and ploidy levels. Chromatin density was estimated from measured metaphase plate width and thickness across eukaryotic species combined with known genome size (Methods; Fig. 1c). We also measured DNA density in RPE1 cells of different ploidy (see comment 5 for details). Analysis of effective metaphase plate chromatin density showed conservation of this parameter across eukaryotes, supporting the assumptions underlying the observed scaling relationships.

"Previous analyses of condensed metaphase chromosomes revealed a remarkably uniform chromatin density across human chromosomes and a subset of eukaryotic species (~90 Mbp μm^{-3})[27–29]. To assess whether this constancy extends to the scale of the entire metaphase plate, we estimated plate volume from measured width and thickness across diverse eukaryotes (Methods). Plate volume scaled with genome size according to a power law with an exponent of 0.96 ($R^2 = 0.91$, $p = 10^{-13}$; Fig. 1e), indicating a near-linear increase in plate volume with chromatin amount. Accordingly, effective metaphase plate density (6.27±0.99 Mbp μm^{-3}) was roughly

constant across genome sizes (Extended Data Fig. 1e). The lower effective density of the whole metaphase plates relative to isolated chromosomes[27–29] likely reflects inclusion of inter-chromosomal spaces in plate volume estimates. Plate shape was likewise similar across eukaryotes, with a width to thickness aspect ratio of 1.89 ± 0.59 (Extended Data Fig. 1f, g). Together, these results indicate that metaphase plate density and shape are conserved across species."

We have also shown that metaphase plate thickness follows similar power-law scaling with genome size across eukaryotes, as metaphase plate width (see comment 9 for details).

The reviewer: *15. The authors stated: "Based on these results, we conclude that substantially larger inter-chromosome pushing forces impede mitosis." Does the spindle fully recover its shape after compression is released? Is the recovery dependent on the duration or strength of compression? If the spindle was distorted or the DNA damaged during compression, that might explain the observed delay in metaphase. In such cases, did they observe any chromosome mis-segregation? Specifically, from Fig. 6i: can the authors separate the cells that return to normal from those that do not, and check mis-segregation frequency?*

The authors: We thank the reviewer for these insightful questions. We have already quantified how spindle shape changes upon release from compression (Figure 4 and Extended Data Fig. 7) and found that spindle morphology only partially recovers shortly (1 min) after release. Regarding whether the extent of recovery depends on the duration or strength of compression, we have now plotted the decrease in spindle height during compression against the increase in height in a more prolonged period (5 minutes after release), combining data from both RPE-1 and U2OS control cells. The findings are now incorporated into Results:

"When compression was relieved, the spindle and metaphase plate partially regained their original widths in both cell types (Extended Data Fig. 7c), similar to spindles in previous studies[66,68], irrespective of the extent of height reduction, which ranged from 2 to 12 µm (Extended Data Fig. 7d)."

We were unable to quantify chromosome mis-segregation in cells after release, as they were stained with a DNA dye that itself induces chromosome mis-segregation during anaphase, but without interfering with earlier mitotic processes (Rajendraprasad et al., 2023.). However, we now acknowledge that spindle distortion and the induction of DNA damage could contribute to the observed delay.

"Although extended mitotic duration could result from physical damage to chromosome attachments during compression, this effect is likely minimal, as the majority of compressed cells eventually progressed to anaphase (Extended Data Fig. 7j)."

The reviewer: M*inor remarks*

a) In the text, please pay attention to the terms used-chromosome number, genome size, nuclear area, amount of chromatin, and DNA content. Define each term clearly and specify the assumptions made regarding their relationships.

The authors: Following the referee's suggestion, we excluded the terms *DNA content* and *genome amount* since they both refer to genome size when discussing organisms and amount of chromatin when discussing single cells. We have additionally replaced the term chromosome number with ploidy when interpreting polyploidization experiments.

The reviewer: *b) Please add statistical correlation directly in the graph of figure 1. Why is the statistical correlation analysis different between 1d and e?*

The authors: We have now included the statistical correlations directly in the graphs of Figure 1 and Extended Data Fig. 1. The statistical analysis itself remained the same, as it was in the original manuscript. However, we have now incorporated all details of the analysis into the caption of Figure 1.

The reviewer: *c) Figure 2b: why does cell number different between up and bottom? Also the number of cells analysed for this figure is quite limited. What is the limiting factor?*

The authors: We thank the reviewer for noticing this issue. The number of cells shown in Figure 2 differed between the upper and lower panels because they were obtained from different experiments. The number of analyzed cells was lower in some cases due to inefficient polyploidization (see comment 3 for details). To improve the efficiency of polyploidization, which had limited the number of analyzable cells, we developed a new polyploidization protocol (see comment 3). Using this improved protocol, we obtained a larger number of cells (exceeding 100 per ploidy group). These new data are now presented in Figure 2b, while the previous data have been moved to Extended Data Fig. 2.

The reviewer: *d) The figures could be better organized. For example, Fig. 2c should come before Fig. 2d, and Fig. 4e should be placed alongside the corresponding experimental analysis.*

The authors: We have now reorganized this figure according to the reviewer's suggestion.

The reviewer: *e) Figure 2h: the MUG image is not very convincing and the cell number is very low (~12). It should be replaced by a clearer image and the sample analysed increase extensively.*

The authors: We agree with the reviewer. We have substantially increased the number of cells in the MUG experiments by approximately threefold. Additionally, the MUG image has been replaced with a clearer representation. The text has been updated according to new results.

"Fourth, we reduced the amount of chromatin in the metaphase plate and disrupted chromosome structure by inducing mitosis with an unreplicated genome (MUG)[51] in the U2OS osteosarcoma cell line (Fig. 2h). In MUG cells, where centromeres are aligned on the spindle but most of the uncondensed chromatin is displaced in the cytoplasm, spindles were 31% thinner without changes in their length or cell size (Fig. 2i; Extended data Fig. 5d, e; note that the metaphase plate width cannot be determined due to uncondensed chromatin). These experiments indicate that the amount of chromatin and its mechanical properties determine spindle width."

The reviewer: f) *In Fig. 3e, add the line of w=15 µm (yellow) to facilitate comparison across conditions.*

Following the referee's suggestion, we have added the yellow line to Fig. 3e (now Fig. 3d) and also other lines that correspond to different plate width values.

The reviewer: g) *Moreover, although the authors suggest that chromosome number is not central, they later show that plate width scales with interphase area /chromosome number (Fig. 2), which contradicts this claim.*

The authors: We thank the referee for pointing out the importance of chromosome number as a possible determinant of the metaphase spindle width. We now acknowledge that in experiments we cannot untangle contributions of chromosome number and genome size (see response to minor comment a). In the new Figure 3, which shows results of theoretical modeling, we now varied both the genome size and the chromosome number in a way that allowed us to disentangle these two contributions. We added these new theoretical results together with the comparison with experiments:

"Motivated by our experiments on altered ploidy and chromosome structure (Fig. 2), we use our model to explore how chromosome number, size, and stiffness affect the inter-chromosome pushing forces and the resulting spindle shape. In an initial step to describe a baseline spindle, our model reproduces a typical spindle shape and predicts only slight deformation of chromosomes (Fig. 3a), for the known stiffness of chromatin and microtubules[60,61]. We next alter the chromosome number while keeping constant either the volume of individual chromosomes or their total volume. In the case of constant volume of individual chromosomes, where an increased number of chromosomes corresponds to the increased total amount of chromatin, the model predicts a power-law scaling of the metaphase plate with an exponent of 0.43 (Fig. 3b). In contrast, in the case of constant total volume of chromosomes, where individual chromosomes become smaller as their number increases, the model predicts a roughly constant metaphase plate width except for narrower plates at small chromosome numbers (Fig. 3c). Thus, spindle shape is set primarily by the total chromosome volume rather than by their number.

To test the model's predictions, we directly compare them with the experimental results on different ploidy. Measurements of spindles in cells with altered ploidy from haploid to diploid to tetraploid (Fig. 2, Extended Data Fig. 2 and 5, Extended Data Table 2), and in oocytes that naturally change from diploid to haploid state (Extended Data Table 2), follow the model's prediction that doubling of chromosome number results in a 35% wider metaphase plate (Fig. 3b). To test the case of constant total volume of chromosomes, we compared 4 species that have a similar genome size (5–8 Gbp), but a substantially different chromosome number (6–46, Extended Data Table 1). These species have a similar metaphase plate width, in agreement with a roughly constant width predicted by the model (Fig. 3c). Thus, irrespective of the way the chromosome number was altered experimentally, and even when comparing different species, the experimental data agree with the model's prediction that the spindle shape is determined by the total chromosome volume."

Reviewer #3 (Remarks to the Author):

The reviewer: *In this very interesting paper, the authors measure the scaling between the genome size and the metaphase plate width, and explain the scaling with a mechanical model based on the idea that the chromosomes are compressed by mechanical forces. The reported scaling is novel, but I find the model completely confusing and unconvincing, and the scaling is so simple that it probably could be explained by several other ideas.*

The authors: We thank the reviewer for the constructive comments. In response to Reviewer #3, we have substantially revised the description of the model to better justify the choice of components and parameters. Moreover, we extended the model to include non-spherically shaped chromosomes, explored the stability of the metaphase plate and how scaling depends on the physical parameters, including the relationship between the chromosome volume and genome size:

- We generalized chromosome geometry by modeling it as a prolate spheroid and expanded the Methods section "Inter-chromosome pushing forces" by including these calculations.
- We performed the stability analysis of the spindle and added a new section in Methods.
- We substantially revised the description of the model in the main text and expanded the list of references by citing additional relevant papers.

The reviewer: *1. The 'spheres' - are those individual chromosomes? - because the individual chromosomes are obviously not spherical in the metaphase plate, as is apparent from many published images.*

The authors: We thank the reviewer for this question. To address this issue, we explored a generalized chromosome geometry by modeling it as a prolate spheroid, which resulted in a similar scaling behavior as when spheres were used. This is now included in the revised Methods section under "Inter-chromosome pushing forces" and in the main text. Along with testing chromosome geometry, we also explored how scaling depends on the remaining physical parameters and the relationship between the chromosome volume and genome size:

"The scaling predicted by the model is robust to changes in the number and physical properties of microtubules and chromosomes (Extended Data Fig. 8e; Methods). Moreover, the scaling does not depend on chromosome shape: incorporating a more realistic prolate spheroidal chromosome geometry produces similar scaling behavior (Extended Data Fig. 8e; Methods). In contrast, the scaling directly depends on the relationship between genome size and chromosome volume. When this relationship is nonlinear, the scaling exponent changes markedly (Extended Data Fig. 8f). The model therefore suggests that the experimentally observed scaling exponent of 0.33 arises from a linear relationship between chromosome volume and genome size."

The reviewer: 2. *It seems that in the model the chromosomes are pressed centripetally inward toward the spindle axis, correct? But then this force is generated by MT pulling? It does not look like this pulling force was considered in the MT elastic rod equation. If MTs are pulling, why do they have the curved shape? What are the boundary conditions at the poles and on chromosomes? Some MTs are KT-MTs, some are chromosomal. The former are supposed to be pulling, the latter - pushing. Comments on this complication?*

The authors: We thank the referee for asking for more clarification on this point. There are no pulling forces in our model, and the chromosomes are pressed centripetally inward because of elasticity of microtubules, which in turn are being constrained at the poles. We have substantially revised the description of the model in the main text to better explain how the forces are balanced:

"To directly test the chromosome crowding hypothesis and estimate the resulting inter-chromosome pushing forces, with the ultimate aim of explaining the spindle scaling law, we introduced a physical model of the spindle. We chose a simple approach where the spindle is composed of chromosomes and microtubules, while omitting proteins unrelated to the chromosome crowding hypothesis, such as microtubule-associated proteins (MAPs). We model each chromosome as a sphere because this is the simplest geometric object characterized solely by its radius, and we use the sphere volume as a proxy for the amount of chromatin (Fig. 3a, and Box 1). In our model, the chromosomes are compressible in response to forces, based on previous measurements

of chromosome elasticity[59]. They are attached to microtubules and positioned in the spindle midplane, representing the metaphase plate. The microtubules, which extend between the spindle poles, push the chromosomes inward towards the spindle axis through their bending forces (Fig. 3a). In response, chromosomes exert inter-chromosome pushing forces as they squeeze against one another. Together, this balance of microtubule bending forces and inter-chromosome pushing forces defines the mechanical equilibrium that sets the chromosome positions and thereby the width of the metaphase plate and the overall spindle architecture (Box 1, Methods)."

The boundary conditions at the pole are described in Methods: "Experiments show that microtubule bundle pivots around the spindle pole under external force[87], which we include in the model by assuming a freely joint condition $M_{i,left} = M_{i,right} = 0$."

The reviewer: 3. *The main problem: if indeed in the model the chromosomes are pressed centripetally inward toward the spindle axis, why would not the metaphase plate buckle? - why would not the inner chromosomes be pushed out away from the equator?*

The authors: We thank the referee for pointing out this important issue. We have performed the stability analysis of the spindle, and added the results in the main text:

„To test under what conditions the inter-chromosome pushing forces are large enough to displace the chromosomes from the metaphase plate, we performed stability analysis of the metaphase plate in the extended model where chromosomes are aligned at the spindle midplane by an elastic spring (Methods). We obtain that chromosome alignment at the metaphase plate is stable if the spring is stiff enough, which is the case for diploid human cells (Extended Data Fig. 2f-i). In contrast, in cells with higher ploidies, the metaphase plate can become unstable for the same spring stiffness, in agreement with the experimentally increased thickness of the metaphase plate (Extended Data Fig. 2f-i)."

To describe the calculations, we added a new section in Methods:

"*Stability analysis of the obtained spindle shape.* In our model, chromosomes are constrained to align at the metaphase plane, whereas in living cells they can be displaced from it. We therefore extend the model by introducing a force directed toward the metaphase plane that still allows such displacements. We perform a stability analysis for the equilibrium solution by calculating the second variation of the energy functional and evaluating under which condition it has a positive value.

To describe a centering force that positions chromosomes, we extend the energy functional in Eq. (M19) by adding an extra term,

$$V_{\mathrm{center}}(\mathbf{r}_{i,\mathrm{chr}}) = \frac{K}{2}\left(\hat{\mathbf{x}} \cdot \mathbf{r}_{i,\mathrm{chr}} - \frac{L}{2}\right)^2, \qquad \text{(M20)}$$

which describes a harmonic centering potential. Here, parameter K denotes strength of centering potential. To calculate the second variation of the energy functional, we consider variation of shapes of microtubule bundles, as well as the variation of spheres' positions. We add a variation to the unperturbed microtubule bundle shape, $\mathbf{r}_i(x) + \epsilon\eta_{y,i}(x)\hat{\mathbf{y}} + \epsilon\eta_{z,i}(x)\hat{\mathbf{z}}$. Here, $\eta_{y,i}$ and $\eta_{z,i}$ are arbitrary functions whereas ϵ is any number close to zero. We also add a variation to the unperturbed chromosome position, $\mathbf{r}'_{i,\mathrm{chr}} = \mathbf{r}_i(x'_{i,\mathrm{chr}}) + \epsilon\eta_{y,i}(x'_{i,\mathrm{chr}})\hat{\mathbf{y}} + \epsilon\eta_{z,i}(x'_{i,\mathrm{chr}})\hat{\mathbf{z}}$, where $x'_{\mathrm{chr}} = L/2 + \epsilon\eta_{x,i}$ is its perturbed x-coordinate.

We expand the position of the spheres in Taylor series up to the second order, since this is the highest order contributing to the second variation of the energy functional. We obtain

$$\mathbf{r}'_{i,\mathrm{chr}} = \mathbf{r}_{i,\mathrm{chr}} + \left(\epsilon\eta_{y,i}\left(\frac{\mathrm{L}}{2}\right) + \epsilon\frac{dy_i}{dx}\bigg|_{\frac{L}{2}}\eta_{x,i} + \frac{1}{2}\epsilon^2\frac{d^2y_i}{dx^2}\bigg|_{\frac{L}{2}}\eta_{x,i}^2 + \epsilon^2\frac{d\eta_{y,i}}{dx}\bigg|_{\frac{L}{2}}\eta_{x,i}\right)\hat{\mathbf{y}} + \left(\epsilon\eta_{z,i}\left(\frac{\mathrm{L}}{2}\right) + \epsilon\frac{dz_i}{dx}\bigg|_{\frac{L}{2}}\eta_{x,i} + \frac{1}{2}\epsilon^2\frac{d^2z_i}{dx^2}\bigg|_{\frac{L}{2}}\eta_{x,i}^2 + \epsilon^2\frac{d\eta_{z,i}}{dx}\bigg|_{\frac{L}{2}}\eta_{x,i}\right)\hat{\mathbf{z}}. \tag{M21}$$

For the variation of the microtubule bundle shape we assume that it is described by functions which obey conditions $\eta_{y,i}(L/2) = 0$ and $\eta_{z,i}(L/2) = 0$. This allows us to obtain an asymmetric microtubule bundle shape for chromosomes displaced from the metaphase plane.

The second order variations of Eq. (M19), together with centering potential Eq. (M20), will result with changes in three terms, representing microtubule bending, inter-chromosome pushing forces, and centering force, $\delta^2(V + V_{\mathrm{center}}) = \delta^2_{\mathrm{bend}} + \delta^2_{\mathrm{chr}} + \delta^2_{\mathrm{center}}$. The respective terms are given by:

$$\delta^2_{\mathrm{bend}} = \frac{1}{2}\epsilon^2\kappa\sum_{i=1}^{N}\int_0^L\left(\frac{d^2\eta_{y,i}}{dx^2}\right)^2 + \left(\frac{d^2\eta_{z,i}}{dx^2}\right)^2 dx \tag{M22a}$$

$$\delta^2_{\mathrm{chr}} = -\frac{1}{2}\epsilon^2 c\sum_{i=1}^{N}\sum_{j>i}^{N}\frac{B_{ij}}{\Delta_{ij}}h_{ij}^{3/2} + \epsilon^2\delta^2_{0,\mathrm{chr}} \tag{M22b}$$

$$\delta^2_{\mathrm{center}} = \frac{1}{2}\epsilon^2 K\sum_{i=1}^{N}\eta_{x,i}^2 \tag{M22c}$$

In the second variation of energy, displacement from the metaphase plane can appear in the case of possibly negative first term in Eq. (M22b). Thus, we decided to write this equation by separating the term with negative value from the term $\epsilon^2\delta^2_{0,\mathrm{chr}}$ with positive value. We also use the shorthands $\Delta_{ij} \equiv |\mathbf{r}_i - \mathbf{r}_j|$, and

$$B_{ij} \equiv \left(\eta_{x,i} - \eta_{x,j}\right)^2 + \left(\eta_{x,i}^2\frac{d^2y_i}{dx^2}\bigg|_{\frac{L}{2}} - \eta_{x,j}^2\frac{d^2y_j}{dx^2}\bigg|_{\frac{L}{2}} + 2\frac{d\eta_{y,i}}{dx}\bigg|_{\frac{L}{2}}\eta_{x,i} - 2\frac{d\eta_{y,j}}{dx}\bigg|_{\frac{L}{2}}\eta_{x,j}\right)\left(y_i\left(\tfrac{L}{2}\right) - y_j\left(\tfrac{L}{2}\right)\right) + \left(\eta_{x,i}^2\frac{d^2z_i}{dx^2}\bigg|_{\frac{L}{2}} - \eta_{x,j}^2\frac{d^2z_j}{dx^2}\bigg|_{\frac{L}{2}} + 2\frac{d\eta_{z,i}}{dx}\bigg|_{\frac{L}{2}}\eta_{x,i} - 2\frac{d\eta_{z,j}}{dx}\bigg|_{\frac{L}{2}}\eta_{x,j}\right)\left(z_i\left(\tfrac{L}{2}\right) - z_j\left(\tfrac{L}{2}\right)\right) \tag{M23}$$

Because the second variation of energy functional of our system results with a very complex solution, we decided to find a lower limit on centering spring and flexural rigidity and compare them with biologically relevant values. We estimate the lower bound in Eq. (M23) by using Young's inequalities,

$$\frac{d\eta_{y,i}}{dx}\bigg|_{\frac{L}{2}}\eta_{x,i}\left(y_i\left(\frac{L}{2}\right) - y_j\left(\frac{L}{2}\right)\right) \le \frac{1}{2\xi^2}\frac{d\eta_{y,i}}{dx}\bigg|_{\frac{L}{2}}^2\left(y_i\left(\frac{L}{2}\right) - y_j\left(\frac{L}{2}\right)\right)^2 + \frac{\xi^2}{2}\eta_{x,j}^2 \tag{M24a}$$

$$\frac{d\eta_{z,i}}{dx}\bigg|_{\frac{L}{2}}\eta_{x,i}\left(z_i\left(\frac{L}{2}\right) - z_j\left(\frac{L}{2}\right)\right) \le \frac{1}{2\zeta^2}\frac{d\eta_{z,i}}{dx}\bigg|_{\frac{L}{2}}^2\left(z_i\left(\frac{L}{2}\right) - z_j\left(\frac{L}{2}\right)\right)^2 + \frac{\zeta^2}{2}\eta_{x,j}^2 \tag{M24b}$$

$$\eta_{x,i}\eta_{x,j} \le \frac{\eta_{x,i}^2}{2} + \frac{\eta_{x,j}^2}{2}. \tag{M24c}$$

These inequalities hold for any choice of parameters $\xi, \zeta \neq 0$. Further, we apply the Poincaré inequality for the function with fixed ends[105],

$$\left.\frac{d\eta_{y,z,i}}{dx}\right|_{\frac{L}{2}}^2 \le \frac{L}{12}\int_0^L \left(\frac{d^2\eta_{y,z,i}}{dx^2}\right)^2 dx, \tag{M25}$$

which is used to calculate Eqs. (M24a) and (M24b). Further, we combine Eqs. (M22), (M23), and (M24) which results in a lower bound of the second variation of energy functional. We calculate in which case the sum of the factors that multiply $\frac{L}{12}\int_0^L \left(\frac{d^2\eta_{y,z,i}}{dx^2}\right)^2 dx$ and $\eta_{x,i}^2$, and appear in both δ_{bend}^2 and negative term in δ_{chr}^2, vanishes. In the first case, we obtain an estimate for lower bound on flexural rigidity,

$$\kappa_{\mathrm{lb}} = \frac{L}{12}\max_i \max\left(\sum_{j\neq i}\frac{F_{ij}}{\Delta_{ij}}\frac{\left(y_i\left(\frac{L}{2}\right) - y_j\left(\frac{L}{2}\right)\right)^2}{\xi^2}, \sum_{j\neq i}\frac{F_{ij}}{\Delta_{ij}}\frac{\left(z_i\left(\frac{L}{2}\right) - z_j\left(\frac{L}{2}\right)\right)^2}{\zeta^2}\right), \tag{M26}$$

whereas in the second case we obtain an estimate for lower bound on strength of centering potential,

$$K_{\mathrm{lb}} = \max_i \sum_{j\neq i}\frac{F_{ij}}{\Delta_{ij}}\left(\xi^2 + \zeta^2 + 2 + \left|\left.\frac{d^2 y_i}{dx^2}\right|_{\frac{L}{2}}(y_i - y_j)\right| + \left|\left.\frac{d^2 z_i}{dx^2}\right|_{\frac{L}{2}}(z_i - z_j)\right|\right) \tag{M27}$$

In Eqs. (M26) and (M27) we define $F_{ij} = |\mathbf{F}_{ij}|$. By evaluating Eqs. (M26) and (M27) we obtain a lower limit for microtubule flexural rigidity and centering potential which we use to calculate stability of the metaphase plate.

To determine the stability conditions for chromosomes aligned in the metaphase plane, we estimate lower bounds on the flexural rigidity and the centering potential strength by evaluating Eqs. (M26) and (M27) using parameters taken from Fig. 3. The central sphere experiences the largest inter-chromosome pushing forces from its neighbors, with $F_{ij} \approx 50$ pN (see Fig. 3d). Because the spheres are only slightly deformed, the distance between their centers can be approximated as twice the radius, $\Delta_{ij} \approx 2.5$ μm. Furthermore, the curvature of the central bundle vanishes $\left.\frac{d^2 y_i}{dx^2}\right|_{\frac{L}{2}}, \left.\frac{d^2 y_i}{dx^2}\right|_{\frac{L}{2}} = 0\ \mu\mathrm{m}^{-1}$. For the arbitrary constants in Eqs. (M26) and (M27), we choose $\xi = 1$ and $\zeta = 1$. For this choice of parameters, lower bounds on flexural rigidity and centering potential strength are $\kappa_{\mathrm{lb}} = 450\ \mathrm{pN\mu m^2}$ and $K_{\mathrm{lb}} = 480\ \mathrm{pN\mu m^{-1}}$, respectively. The value of flexural rigidity parameter for microtubule bundles is calculated from value for a single microtubule, $\kappa = 900\ \mathrm{pN\cdot\mu m^2}$ [106,107]. This value is indeed above the lower bound $\kappa > \kappa_{\mathrm{lb}}$. Centering potential strength, however, is not measured directly and thus we estimate its value from measured kinetochore forces and displacement from the metaphase plate. Owing that forces acting on chromosomes are several hundreds of pN[62–64] and typical displacement of chromosome is about 1 μm[108], estimated centering potential strength is also several hundreds of pN/μm and thus comparable with lower bound K_{lb}."

The reviewer: *4. Presumably, as the forces are MT-related, the scaling should depend on the MT number. Was this researched?*

The authors: We thank the reviewer for this suggestion, which we explored in the new Extended Data Figure 8e. Please see also our response to point 1 above.

The reviewer: *Additional questions and comments:*

Are the spindles cylindrically symmetric? - does the metaphase plate have a cylindrical shape? If not, I am not sure what was measured in the paper, and in adherent cells (and also in tissue cells) spindles are rarely cylindrically symmetric.

The authors: We appreciate these comments. We have now explained in the Methods how metaphase plate dimensions were measured and why we approximate metaphase plate as a cylinder based on extensive data that was previously published:

"In a cross-section of the metaphase plate, chromosome arms stick out, resulting in an irregular shape of the plate. We estimated the effective volume of the metaphase plate by approximating the shape of the plate with a cylinder. This approach allowed us to analyze published experimental data, many of which lack information about the three-dimensional shape of the spindle. Approximation of the plate shape with a cylinder was validated in previous studies on diverse systems including: mouse embryonic stem cells, Ptk2 cells[22], human HeLa and RPE1[32,72,114], and multiple Ichthyosporea species[115]. We estimated the metaphase plate volume in different eukaryotic species from the measured width (W) and thickness (T) using the formula for a cylinder: $V = \pi \cdot \left(\frac{W}{2}\right)^2 \cdot T$. Metaphase plate density across species was calculated as the ratio between genome size and the estimated volume of the metaphase plate."

The reviewer: *In fig 1, it is apparent that the difference between the metaphase plate width and spindle width are just 'rabbit-ears' of the chromosome arms, which are quite obviously not cylindrical... Comments?*

The authors: We agree that the chromosome arms stick out, which we addressed in Methods as described in the previous comment.

The reviewer: *Presumably, one can compute the total chromosome volume from the metaphase plate thickness and width - and then compare it with the nuclear volume - this will be a good sanity check. Was this done? What is the relation between thickness and width?*

The authors: Even though we could not directly answer the first part of this comment because chromosomes undergo extensive compaction during early mitosis driven by condensin, topoisomerase II activity and chromatin re-organization in prophase and prometaphase (Hirano et al., 2016.), and thus the metaphase plate volume is not comparable the interphase nuclear volume, we computed the total chromosome volume from the metaphase plate thickness and width over different species, as well as the relation between thickness and width. This analysis led to interesting results that the metaphase plate density and shape are conserved across eukaryotic species:

"Previous analyses of condensed metaphase chromosomes revealed a remarkably uniform chromatin density across human chromosomes and a subset of eukaryotic species (~90 Mbp μm^{-3})[27–29]. To assess whether this constancy extends to the scale of the entire metaphase plate, we estimated plate volume from measured width and thickness across diverse eukaryotes (Methods). Plate volume scaled with genome size according to a power law with an exponent of 0.96 ($R^2 = 0.91$, $p = 10^{-13}$; Fig. 1e), indicating a near-linear increase in plate volume with chromatin amount. Accordingly, effective metaphase plate density (6.27±0.99 Mbp μm^{-3}) was roughly constant across genome sizes (Extended Data Fig. 1e). The lower effective density of the whole metaphase plates relative to isolated chromosomes[27–29] likely reflects inclusion of inter-chromosomal spaces in plate volume estimates. Plate shape was likewise similar across eukaryotes, with a width to thickness aspect ratio of 1.89 ± 0.59 (Extended Data Fig. 1f, g). Together, these results indicate that metaphase plate density and shape are conserved across species."

The reviewer: *Lastly, a paper on spindle scaling cannot omit citing the following two fundamental papers (I am, by the way, not one of these papers' co-authors and have no hidden agenda :)) and discussing what your study implies for their results:*

Stoichiometric interactions explain spindle dynamics and scaling across 100 million years of nematode evolution. Reza Farhadifar et al, eLife 2020

Scaling, selection, and evolutionary dynamics of the mitotic spindle. Reza Farhadifar et al., Curr Biol. 2015 Mar 16;25(6):732-740. doi: 10.1016/j.cub.2014.12.060. Epub 2015 Feb 12.

Similarly, this review lists all possible mechanisms of the spindle scaling:

Force and length in the mitotic spindle. Sophie Dumont, Timothy J Mitchison. Curr Biol. 2009 Sep 15;19(17):R749-61. doi: 10.1016/j.cub.2009.07.028.

It'd be good to cite it (again, I am not one of the authors) and discuss.

I realize of course that your focus is not on the spindle length but on the metaphase plate width.

The authors: We thank the reviewer for highlighting these important studies. We have now incorporated them into the manuscript: the first two (Farhadifar et al., 2015, 2020) are cited when introducing the concept of spindle scaling with cell size across species, while the third (Dumont and Mitchison, 2009) is cited in the context of spindle scaling mechanisms during early embryonic development.

"In particular, spindle size is adapted to large variations in cell size during embryonic development within individual species[6], as well as to different cell sizes and genome sizes across species[7–9]. As cells shrink during early embryonic development, spindle size decreases accordingly, regulated by mechanisms that control microtubule nucleation and dynamics in a cell size-dependent manner[10–20]."

The reviewer: *Reviewer #3 (Remarks on Protocol(s)):*

I am not a great expert on experiments, but all seems to be in order. The model, as I wrote, I found confusing - could be just the problem with explaining it better - I know the authors' previous work, and it is excellent.

The authors: We thank the reviewer for the constructive feedback, which helped us to improve the clarity of the model and analysis of the experimental data.

Rebuttal letter NCB-A57858A

Reviewer #1 (Remarks to the Author):

The reviewer:
A. Remarks to the Author:
The authors seem to have done a good job responding to reviewer concerns.

The reviewer:
B. Remarks on code availability:
You cannot expect reviewers to do this kind of work for you. This would be a ton of work!

C. Remarks on Protocol(s):
You cannot ask reviewers to do this much work. Ask GPT5 to review, it will do a good job

The authors: We thank the reviewer for this positive assessment and for their time and effort throughout the review process.

Reviewer #2 (Remarks to the Author):

The reviewer:
A. Remarks to the Author:
The authors did an impressive job addressing all the comments, and the final manuscript is now very solid. I have just one final note regarding comment #7: for clarity, I suggest that the authors include in the Discussion the hypothesis explaining the difference between adaptive hypotetraploid clones and those experimentally induced. This could help justify the lack of difference in metaphase plate width/thickness between hypotetraploid and tetraploid cells, despite their differing genome densities

The authors:
We thank the reviewer for this positive evaluation and helpful final suggestion. In response, we have revised the Discussion section:

"We found that adapted cells and their spindles are often larger than freshly generated cells of the same increased ploidy (Fig. 2), and that this larger size correlates with a distinct transcriptomic signature (Extended Data Fig. 4). Larger spindles could also explain the wider metaphase plates observed in adapted cells, given the weak coupling between spindle length and metaphase plate width. Alterations in cell and spindle size may therefore represent a general mechanism of cellular adaptation to polyploidization[97]."

Reviewer #3 (Remarks to the Author):

The reviewer:
A. Remarks to the Author:
The authors properly addressed all critiques and questions, and the paper is ready for acceptance.

B. Remarks on code availability:
The equations the authors use are quite easy and transparent and are explained well in the SI.

C. Remarks on Protocol(s):
I am not an expert on the experimental protocols.

The authors:
We thank the reviewer for this positive assessment and for their constructive feedback throughout the review process.
