# Supplementary material for: Power-law scaling of mitotic spindles with genome sizes across eukaryotes is driven by chromosome crowding
Source: Nat Cell Biol. 2026 Oct 9;28(10):1996–2007. doi: 10.1038/s41556-026-02005-8 (PMC13661270; doi:10.1038/s41556-026-02005-8)

Extended data Fig. 4c Source Data

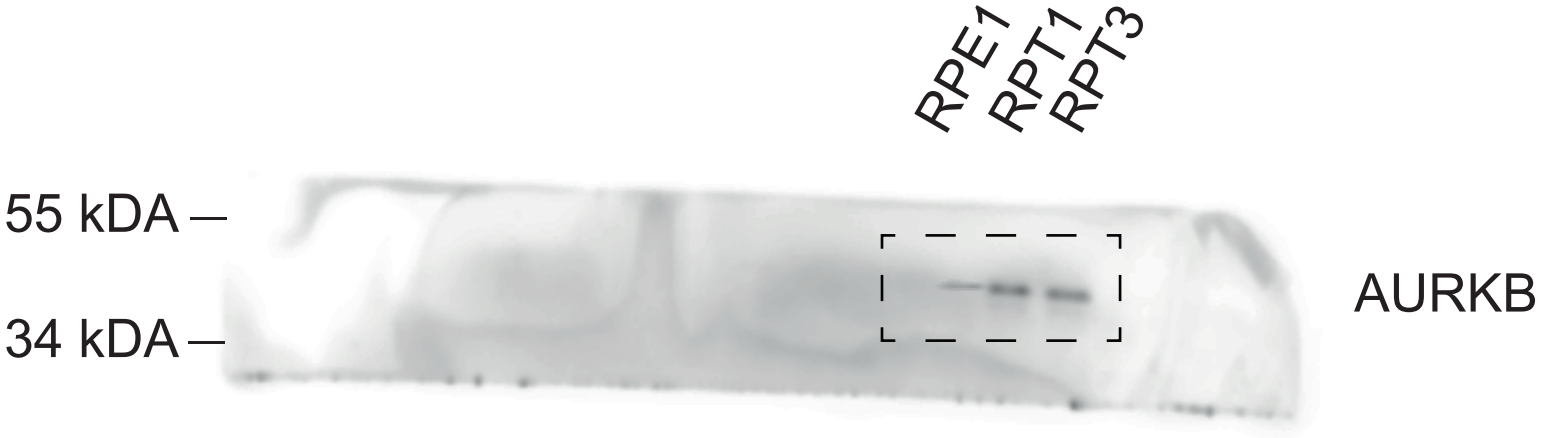

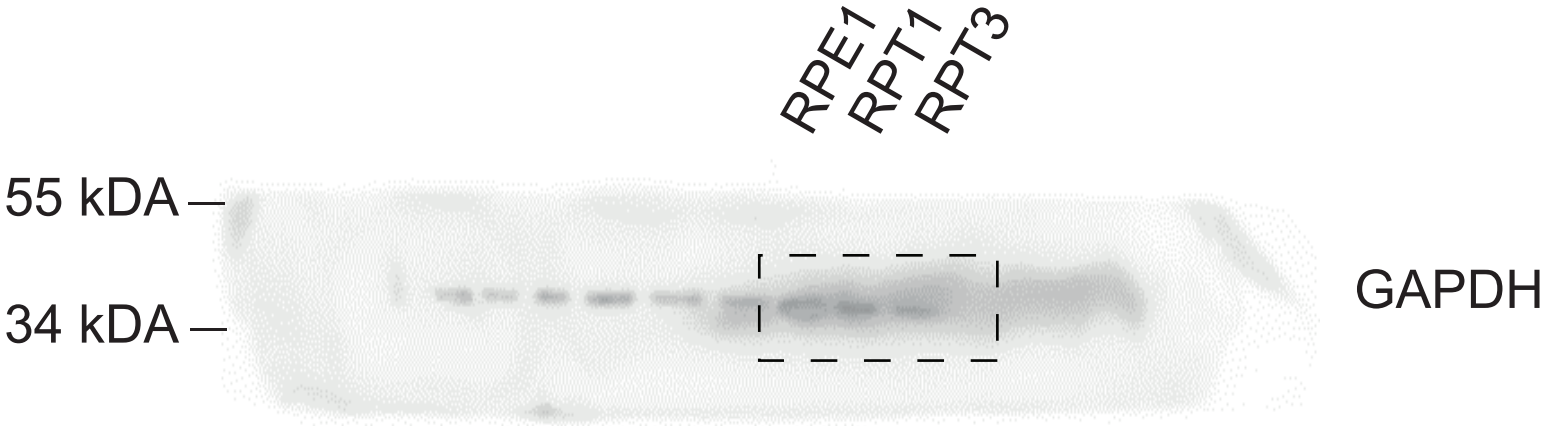

Supplement: Supplementary file 3 [file 41556_2026_2005_MOESM9_ESM.pdf]
